# A Generative Framework for Probabilistic, Spatiotemporally Coherent Downscaling of Climate Simulation

**DOI:** 10.1038/s41612-025-01157-y

**Published:** 2025-07-18

**Authors:** Jonathan Schmidt, Luca Schmidt, Felix M. Strnad, Nicole Ludwig, Philipp Hennig

**Affiliations:** 1 University of Tübingen, Tübingen, Germany; 2 Tübingen AI Center, Tübingen, Germany

**Keywords:** Atmospheric dynamics, Mathematics and computing

## Abstract

Local climate information is crucial for impact assessment and decision-making, yet coarse global climate simulations cannot capture small-scale phenomena. Current statistical downscaling methods infer these phenomena as temporally decoupled spatial patches. However, to preserve physical properties, estimating spatio-temporally coherent high-resolution weather dynamics for multiple variables across long time horizons is crucial. We present a novel generative framework that uses a score-based diffusion model trained on high-resolution reanalysis data to capture the statistical properties of local weather dynamics. After training, we condition on coarse climate model data to generate weather patterns consistent with the aggregate information. As this predictive task is inherently uncertain, we leverage the probabilistic nature of diffusion models and sample multiple trajectories. We evaluate our approach with high-resolution reanalysis information before applying it to the climate model downscaling task. We then demonstrate that the model generates spatially and temporally coherent weather dynamics that align with global climate output.

## Introduction

Numerical weather and climate simulations based on discretized solutions of the Navier-Stokes equations are fundamental to understanding large-scale weather patterns, climate variability, and climate change. State-of-the-art numerical weather prediction (NWP) models, which primarily focus on atmospheric processes, can accurately resolve small-scale dynamics within the Earth system, providing fine-scale spatial and temporal weather patterns at resolutions on the order of kilometers^1^. However, the substantial computational resources required for these models render them impractical for simulating the extended time scales of multiple years and decades necessary to assess climatic changes. Moreover, even with substantial computational investment, global high-resolution models can still exhibit systematic biases and may fail to accurately reproduce observed climatic trends^2^. In contrast, Earth system models (ESMs), such as those included in the CMIP6 project^3^, incorporate a broader range of processes—including atmospheric, oceanic, and biogeochemical interactions—while operating on coarser spatial scales. Typical grid resolutions for ESMs are ~1°, equivalent to around 100 km. This coarse resolution limits the ability of ESMs to fully capture small-scale processes. Key processes necessary to assess regional impact, for example, on wind turbines—such as local wind turbulence—occur at spatial and temporal scales that are too fine to be explicitly resolved in ESMs. Consequently, ESM data cannot be directly employed to evaluate changes at fine spatial scales, limiting their utility for localized impact assessment and decision-making.

Downscaling aims to provide regional climate information by estimating small-scale processes from coarse simulations of global models. Existing approaches to bridge this scale gap can be categorized into dynamical and statistical downscaling^4^. Dynamical downscaling employs high-resolution regional climate models (RCMs) that are nested within coarser global climate models (GCMs) to provide detailed projections for specific regions^5,6^. Biases from the driving global models can be inherited by the RCMs, potentially limiting the accuracy of the downscaled results^7^. Additionally, the high computational cost of running RCMs restricts their use primarily to regional studies. Statistical downscaling methods use regression or weather generators^8^, and, more recently, emulators based on machine learning (ML) techniques. In statistical downscaling, a functional or statistical relationship between large-scale climate variables (from GCMs) and local observations is established. Based on this relationship, statistical methods infer the local information from the coarse simulation, with ML-based approaches aiming to capture the mapping from coarse to fine-scale climate by learning the relationships from data^9–12^. These models are usually computationally less expensive. However, not explicitly encoding physical laws can make them less robust in regions of low data density, risking physical inconsistencies.

One challenge that comes with downscaling arises from the fact that numerical models are inherently imperfect representations of the climate system. While ESMs are designed to generate accurate multi-decadal summary statistics, locally, the simulations differ from historical observational datasets. Climate models contain inaccuracies in parameterization, simplified process representations, and uncertainties in the initial state of the system^13^. Even in the hypothetical case of perfect models, i.e., without epistemic uncertainty, forecasts are not deterministic due to the chaotic nature of the atmosphere. These variations lead to substantial discrepancies between models. Choosing a model, therefore, becomes a critical factor^14,15^. Aside from other climate model biases^16^, internal variability of the climate system leads to differences between projected and observed climate^17^. Individual climate simulations thus represent only one possible realization of the system with substantial uncertainty remaining. Due to this un-pairedness of ESM outputs and observational data, using supervised ML approaches, which rely on consistent simulation–observation pairs, remains challenging^18,19^. Generative models have recently emerged as a promising solution. This model class is characterized by learning a representation of the training data distribution that allows the generation of novel samples. As self-supervised learning techniques, these models circumvent the need for data–label pairs by working solely on the target (output) distribution. Additionally, through the variability among generated samples, generative models provide structured uncertainty, which the ill-posed nature of most inference problems entails. In particular, diffusion models (DMs)^20–23^ have demonstrated superior performance over earlier approaches such as variational auto-encoders (VAEs)^24^, generative adversarial networks (GANs)^25^, and normalizing flows^26^, particularly for structured data and image generation tasks^27–29^.

**Fig. 1:**
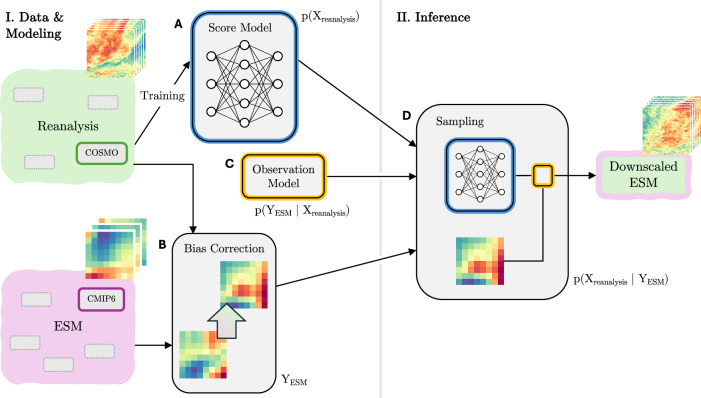
Probabilistic pipeline for spatiotemporal downscaling of multiple variables. This work introduces a probabilistic downscaling framework that jointly predicts fine-scale and spatiotemporally consistent time series for multiple variables from coarse ESM simulations. This schematic outlines the framework. Only one exemplary variable is shown for visual clarity. **A** A score model is trained on sequences of reanalysis data. This is the centerpiece of the diffusion model that learns to reproduce the fine-scale spatial and temporal patterns. Note that ESM simulations are not part of the training process. **B** From any ESM (e.g., CMIP6), select and pre-process an ensemble run (e.g., MPI-HR) for downscaling. A bias-correction step that mitigates distributional deviations between climate output and reanalysis data can be applied as a pre-processing step. **C** This part establishes a relationship between coarse climate simulations and the (fine-scale) output space of the model. The observation model defines how the observed quantity (*Y*_ESM_) is generated---or observed---from the inferred quantity (*X*_reanalysis_). The observation model is key to impose a constraint onto the generative model such that its samples adhere to the established relationship. **D** The model generates time series that preserve the statistics of the coarse climate input. During the generative process, the trained score model (**A**) is conditioned, i.e., the predictions are informed by the conditioning information (**B**), such that they adhere to the relationship established by the observation model (**C**).

In this work, we build on the score-based data assimilation (SDA) framework by Rozet and Louppe^23,30^ to phrase downscaling as a Bayesian inference problem with a generative prior model. By construction, our model performs *joint* spatial and temporal downscaling on multiple variables, introducing stochasticity solely between samples. The predictions are thus coherent across the entire state space, avoiding sampling-induced inconsistencies between time steps and between interrelated atmospheric variables. Furthermore, the model training is separated from the task-specific inference (Fig. 1I, II), which makes the model flexible with respect to its input and the statistical relationship between input and output—allowing, for instance, downscaling climate simulations of varying spatiotemporal resolutions without retraining the model. The components of the algorithmic pipeline are outlined in Fig. 1:

A. The score model, which is the centerpiece of the generative diffusion model, is trained on high-resolution reanalysis data. The score-based diffusion model provides a statistical representation of local weather dynamics. This “*prior*” model gives access to samples from the distribution *p*(*X*_reanalysis_), which represents our prior concept of the output space. Here, the output space includes the spatial region, the target resolution, and learned dynamics patterns for a set of selected atmospheric variables.
B. The coarse ESM input *Y*_ESM_ is pre-processed; in particular, a bias-correction procedure can align the simulation with the reanalysis data in terms of its value distribution.
C. The “*observation model*” *p*(*Y*_ESM_∣*X*_reanalysis_) establishes the functional or statistical relationship between coarse climate output and fine reanalysis data. This assumes the ESM output to be a perfect prognosis^31,32^, based on which local climate is estimated. In the context of inverse problems, the observation model is often referred to as the “forward model”, as it models how the partially observed quantity (*Y*_ESM_) arises from the latent quantity of interest (*X*_reanalysis_)—usually by removing information.
D. Accordingly, the inverse problem aims to predict the (much harder, underspecified) opposite direction: estimating the “*posterior*” *p*(*X*_reanalysis_∣*Y*_ESM_) requires adding information to the incomplete observations by conditioning the prior. In our case, the trained score model is coupled with the observation model to generate samples from the posterior distribution.

Perspectively, the algorithmic framework is versatile and flexible enough to be considered beyond downscaling. In the spirit of foundation models^33–36^, the trained generative model can be combined with other observation models in a similar zero-shot manner to solve various inference tasks on the target output space. Unlike approaches that require training the model directly on the conditioning information, the presented framework allows the formulation of explicit—and varying—functional or statistical relationships between observations and predictions.

On a series of experiments, we demonstrate that the proposed model generates coherent time series of regional climate that are aligned with coarse input. The model predicts local weather dynamics, including extreme events such as winter storms. Sampling from the posterior distribution enables the generation of multiple weather trajectories crucial for assessing the internal variability and uncertainty of the downscaling problem, providing a comprehensive understanding of future weather scenarios. We include a simple quantile-mapping procedure as a pre-processing step to the ESM simulations. Other than that, the ESM simulations are taken as a perfect predictor, and the mismatch to the observational data is not accounted for. We evaluate our downscaling framework on two different GCMs from CMIP6 in order to show that different mismatches between reanalysis and the respective ESM distributions remain. We then demonstrate that our model maintains the coarse, global properties of the respective ESM input while inferring local fine-scale weather patterns.

## Results

The presented downscaling pipeline assumes a statistical relationship between a coarse numerical model prediction and a fine-scale reanalysis product^31,32^. We begin by evaluating the methodological framework in an artificial setup, in which coarsened reanalysis data serve as the perfect prediction^37^ and surrogate the ESM simulations. The coarse data is obtained from spatial area averages and by selecting a subset of the time steps. Concretely, the observation model establishes the following relationship between the downscaled predictions *X* and the coarse input *Y*:

1 $${Y}_{t}^{(i,j)}=\frac{1}{| \overline{i}| \cdot | \overline{j}| }\cdot \sum _{({i}^{{\prime} },{j}^{{\prime} })\in \overline{i}\times \overline{j}}{X}_{t}^{({i}^{{\prime} },{j}^{{\prime} })},$$

where (*i*, *j*) is a single point on the coarse spatial grid, which represents an area of multiple points $\overline{i}\times \overline{j}$ on the fine grid. For example, $\overline{i}$/$\overline{j}$ can be a local neighborhood around *i*/*j* in the longitude/latitude dimension. The observation model in Eq. (1) assumes that the coarse information is provided as a snapshot at time points *t*. In our experiments, one coarse-grid point (*i*, *j*) encompasses a 16 × 16 area on the fine grid, i.e., $| \overline{i}| \cdot | \overline{j}| =1{6}^{2}=256$. Furthermore, the temporal resolution of the coarse grid is 6 h, whereas the fine output time grid is resolved hourly. This setting enables a direct pairing between climate output and reanalysis data, which is not given in reality^37^, allowing for evaluating the model’s predictive performance and uncertainty quantification by comparing it to a ground truth. Having established the validity of the downscaling method on this artificial setup, we will then proceed to downscale climate output, assuming two different ESM simulations from the CMIP6 project to be the perfect predictors.

### Evaluation of predictive distribution and uncertainty calibration

**Fig. 2:**
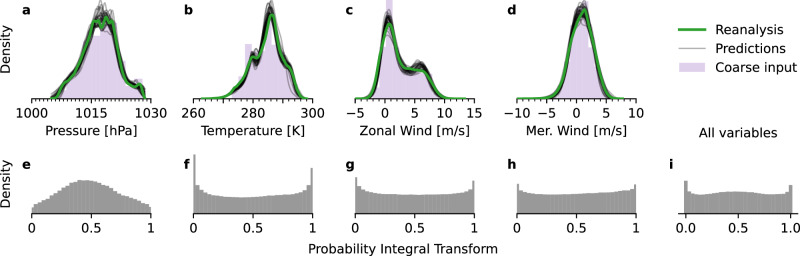
Comparison of value distributions: reanalysis data, coarse input, predictions. For a time range of 49 h, this plot shows that the 1-hourly local predictions, which the model predicted from the coarse 6-hourly input, resemble the reanalysis data closely in distribution. *Top row:* Kernel-density estimations for value distributions of reanalysis data (green) and 30 predictions (black) for each variable separately (**a**–**d**). The prediction model was conditioned on coarse inputs (purple). The predicted samples align with the reanalysis data distribution, which is fully covered by the predictive uncertainty. *Bottom row:* The probability integral transform (PIT) demonstrates the uncertainty calibration of the model: for each variable separately (**e**–**h**) and overall (**i**). A PIT distribution that resembles a standard uniform distribution indicates that the reanalysis data and predictions likely come from the same distribution.

We first evaluate the basic capability of the model to adhere to the established statistical relationship between coarse and fine predictors (cf. Eq. 1) and to preserve the coarse-data value distribution in its predictions. Aggregating the values over temporal and spatial domains, we find that—per variable—the estimated densities of the predictions each align with the reanalysis data. The uncertainty induced by the sample spread covers the reanalysis distribution both near the modes and in regions of low and high quantiles. In particular, the probabilistic model introduces no systematic biases, like distribution shift, a tendency towards over- or under-predicting values, or mismatch in the tail regions. Instead, each sampled prediction captures the spread of the data distribution. Figure 2a–d visualizes these findings using density estimations of the respective value distributions.

To assess the calibration of the predictive uncertainty, we compute the probability integral transform (PIT)^38^ for all values aggregated and for each variable separately. If the resulting distribution (Fig. 2e–i) resembles a standard uniform distribution on the interval [0, 1], it is likely that the samples and the reanalysis data come from the same underlying distribution. We find that both wind-speed components (Fig. 2g, h) are well calibrated. The PIT reveals that the predictions for mean sea-level pressure (Fig. 2e) are slightly underconfident, over-predicting extreme values. For surface temperature (Fig. 2f), the opposite is the case: the predicted distribution is slightly too narrow and under-predicts tail events. Taking the joint distribution of variables together (Fig. 2i), the model is well calibrated.

### Predicting local dynamics from coarse information

We require our downscaling model to augment the scarce information contained in the coarse input by adding nontrivial, local weather patterns and predicting complex temporal and spatial dynamics. As described above, in our experimental setup, a single scalar measurement informs a 6-h window of 16 × 16 fine-grid locations, requiring the model to perform a mapping from a single node to 6 × 16 × 16 = 1536 nodes. Hence, we need to assess whether the model sensibly incorporates prior knowledge, which it learned from data, in order to evaluate the plausibility of the added, generated information.

It is likely that—due to, for instance, varying environmental conditions—the dynamical patterns at distinct locations on the reanalysis grid differ substantially from each other, whereas the coarse-grid aggregation occludes these local variations, motivating the downscaling problem in the first place. The present input-output-paired setup allows us to investigate the true local variations that are lost through aggregation by comparing the reanalysis data at two distant locations within the 16-by-16 area that is encompassed by a single coarse-grid location. Accordingly, we can compare these ground-truth variations with our model predictions to assess whether the model infers local dynamics that align with the reanalysis data.

**Fig. 3:**
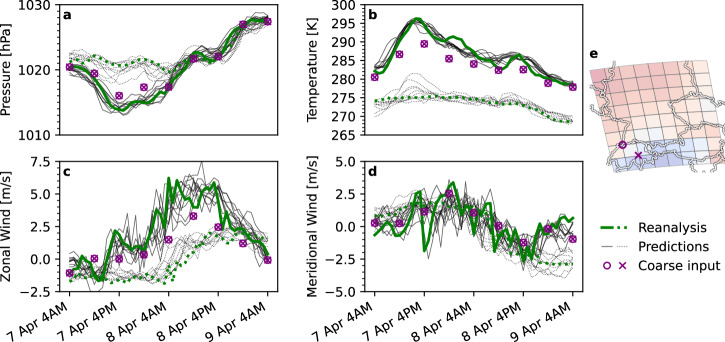
Differences in local dynamics are inferred from coarse-grained observations. This plot compares time series at two locations over 49 h between reanalysis data (green), 30 predictions (black), and coarse input (purple) and shows that the model can accurately extract local dynamics from shared coarse information. Both fine-grid locations were selected to share a single point on the coarse grid (**e**). Each plot **a**–**d** shows one variable. Reanalysis and predicted weather trajectories are shown in solid and dotted lines for both locations, respectively. The conditioning information (purple circles and crosses) is a single value every 6 h that is shared by both locations (**e**), at which the local weather dynamics are inferred. The predicted time series aligns with the reanalysis data at both locations. In particular, the uncertainty obtained through sampling multiple predictions covers the reanalysis data, and the individual samples mirror the local weather trajectories in their respective temporal structure.

In Fig. 3, we demonstrate that our model accurately predicts spatial and temporal variations in weather trajectories at two distinct locations, which share a single spatial observation. For this experiment, we selected two distant fine-grid locations near the Alps, for which we can expect substantial local variations (see purple circle and cross in Fig. 3e). We visualize the time series for the reanalysis data at both locations (solid and dotted green line) and the corresponding downscaled model predictions (solid and dotted black lines) alongside the coarsened input (purple circle and cross), which share a single value at every 6-hourly step. The visualization allows to identify the local variations that are occluded in the spatiotemporal aggregation by comparing the coarse observations (purple) to the fine ground-truth time series. Our model accurately predicts the ground-truth dynamics at both locations from the coarse information. Notably, the distinct features in temporal structure (e.g., smoothness and amplitude) of the predicted time series at both locations align with those found in the corresponding reanalysis data. As an illustrative example, the zonal wind speeds (Fig. 3c) show substantial variability between the two locations—both in their values and their temporal structure. The model captures these variations in its predictions, highlighting its ability to recover local-scale variability lost in the coarse-resolution observations. This is a promising indicator that the downscaling model has learned an accurate representation of local spatial and temporal patterns from the training data, which it blends into the coarse conditioning information to estimate coarsely informed local variations.

### Spatiotemporally consistent weather trajectories: studying a winter storm

**Fig. 4:**
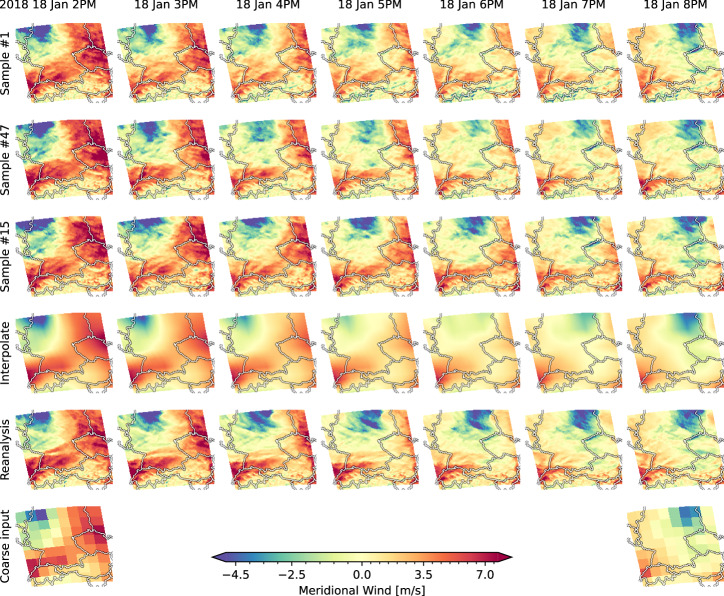
Predicting high-resolution dynamics during a cyclone as an extreme event. This plot shows meridional wind trajectories of three randomly selected model predictions (top three rows), spatiotemporal interpolation (fourth row), reanalysis data (fifth row), and coarse conditioning information (bottom row) during a cyclone (“Friederike”, January 2018). The sign of the wind speed value defines its direction: negative values go southward, and positive values go northward. Time progresses from left to right hourly, starting 2018 January 18 at 02:00 P.M. and ending the same day at 08:00 P.M. Between the first (2:00 P.M.) and the last (8:00 P.M.) visualized time point, no information is provided to the model. The top three rows show how different samples add missing spatial and temporal information, while the interpolation (fourth row) can only spread out the existing, coarse information. Each individual generated trajectory aligns visually with the coarse input, while the variation among the samples captures the uncertainty associated with the inference problem. Notably, the model does not introduce implausible “jumps” from one time step to the next but interpolates with spatially and temporally consistent dynamics.

Statistical downscaling becomes particularly challenging during extreme events, which are rare and fall into the tails of the training distribution. These events often involve highly complex dynamics. However, extreme events are particularly interesting, as they often have the most significant societal and environmental impacts. As Fig. 2 already demonstrated, the downscaling model is capable of accurately predicting high quantiles. Here, we supplement the evaluation of the aggregated value distributions with a qualitative assessment of the spatiotemporal structure of the downscaled predictions during a winter storm. To this end, we consider the time range in which cyclone “Friederike” approached central Europe (including the modeled spatial region) from the west around January 18, 2018. Aside from the time period, the experimental setup remains unaltered from the above sections. Figure 4 exemplary shows the meridional wind speeds during the event. The visualization compares three randomly selected model predictions, a spatiotemporal interpolation of the coarse input, the ground-truth reanalysis data, and the coarse input. The model predictions are coherent in space and time and add different local variations to the coarse observations. A comparison to the spatiotemporal interpolation (Fig. 4, fourth row) highlights the capability of the downscaling model to predict nontrivial fine-scale patterns. Supplementary Fig. 9 visualizes anomalies—differences between the interpolation and (a) downscaled predictions and (b) reanalysis data. This visualization reveals the spatiotemporal patterns, which are lost by aggregating the reanalysis data, and allows a comparison to the disaggregated fine-scale structure predicted by our downscaling model. Additionally, Supplementary Fig. 2 demonstrates a close match of power spectral densities between reanalysis data and model predictions, providing a more quantitative argument.

### Downscaling ESM simulations

**Fig. 5:**
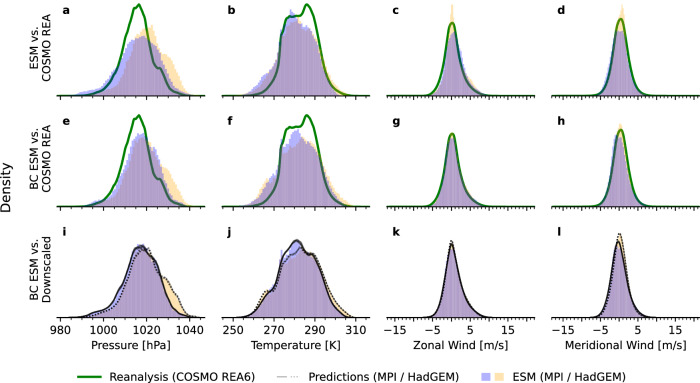
Comparison of value distributions: ESM, de-biased ESM, reanalysis data, predictions. This plot visualizes aggregated value distributions of reanalysis data (green), ESM simulations (two different models: MPI in purple, HadGEM in yellow), and downscaled predictions (black). The *top row* compares the raw, uncorrected ESM simulations with the reanalysis data to visualize the biases in the respective climate distributions. The *middle row* shows the bias-adjusted ESM simulations alongside the same reanalysis data to visualize the effect of the quantile-mapping procedure. The *bottom row* compares the value distribution of the bias-adjusted ESM simulations (same as middle row) with their downscaled counterpart (black). The eight solid/dotted lines correspond to downscaled predictions of the MPI/HadGEM model. Two things are demonstrated: Comparing the *top row* and the *middle row* shows that the distribution mismatch between both ESM ensembles and the reanalysis data (**a**–**d**) is mitigated through the bias-correction step (**e**–**h**). There is some mismatch remaining, likely due to the short evaluation period. Secondly, the *bottom row* shows for each variable separately (**i**–**l**) that the distribution of the downscaled climate trajectories aligns with the coarse model input. The considered time range is the year 2014. The model predicts 1-hourly steps starting January 01 at 06:00 A.M. and ending December 31 at 06:00 A.M. based on the corresponding 6-hourly coarse input. Visualizing two distinct CMIP6 ensembles (MPI and HadGEM) allows a comparison of the distributional mismatch, **a** between the respective climate outputs (purple vs. yellow) and **b** between climate outputs and reanalysis data.

**Fig. 6:**
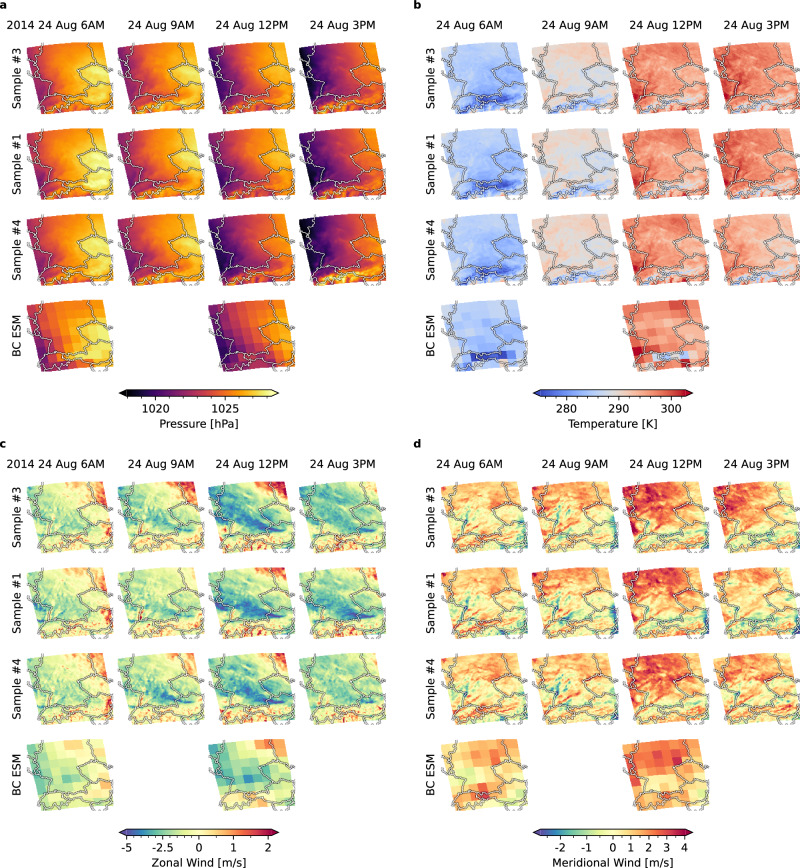
Local climate information through downscaling. The plot shows spatiotemporally downscaled predictions for the MPI-HR ensemble that is part of the CMIP6 simulations. Time progresses from left to right in 3-hourly steps, starting on August 24, 2014, at 06:00 A.M. and ending the same day at 03:00 P.M. The four variables, mean sea-level pressure (**a**), surface temperature (**b**), zonal (**c**) and meridional (**d**) wind-speed components, are downscaled jointly by the model. The top three rows show the progression of three randomly selected samples. The bottom row shows the corresponding conditioning information from the coarse bias-corrected (BC) ESM simulations. Where the observation is blank, the model interpolates in time, without any conditioning information.

The above experiments evaluated the model in a setting that ensures that the large-scale predictor perfectly matches the statistics of the fine-scale reanalysis data. We proceed to downscaling climate-model outputs, using 6-hourly CMIP6 ESM simulations as conditioning information. As in the previous experiments, the four variables considered are downscaled spatially (by a factor of 16 × 16) and temporally (by a factor of 6) to align with the resolution of the reanalysis data. The same statistical relationship (Eq. 1) between coarse input and downscaled output is assumed in this experiment. Downscaling climate-model outputs, however, presents a greater challenge because there is no direct pairing between coarse- and fine-scale climate data, which renders the comparison of our downscaling predictions to a ground truth impossible. The goal of this experiment is to predict local patterns on a fine spatiotemporal grid, assuming two distinct perfect predictors given by two realizations from the distribution of coarse climate simulations. We evaluate whether our downscaling model—while adhering to the statistical relationship imposed between each prediction and different coarse ESM simulations (Fig. 5)—is capable of simultaneously predicting local climate (Fig. 6). Notably, climate model biases are not encoded directly and therefore not addressed by the downscaling model. As motivated by Volosciuk et al. ^39^, downscaling can be separated into, firstly, mitigating climate model biases and, secondly, bridging the gap between coarse and fine grids. We adopt this perspective, thereby focusing almost exclusively on the latter. As a pre-processing step, we apply a per-variable quantile-mapping bias-correction^40^ to the ESM outputs.

Figure 5a–d shows two model ensembles of the four variables from different climate models: the MPI-HR (purple) and the HadGEM model (yellow) for the year 2014 and, in comparison, the reanalysis data distribution (green curve). Comparing the distributions of the two ESMs, it becomes evident that, even in terms of their aggregated-value distribution, climate model outputs can differ significantly from each other and, moreover, each individual ESM differs substantially from the reanalysis data. Figure 5e–h visualizes the effect of the quantile-mapping bias adjustment, which adjusts the marginal distributions of each variable in the ESM simulations to better match those of the reanalysis data. As a result, the bias-corrected ESM simulations more closely align with the reanalysis data (green curve). A comparison of the uncorrected and bias-corrected distributions (Fig. 5a–d vs. e–h) reveals that some residual differences remain. Figure 5i–l visualizes the distribution of eight downscaled predictions (black) for each of the two climate models. The downscaled predictions for MPI-HR (solid lines) and HadGEM (dotted lines) closely match the value distributions of their respective bias-corrected ESM simulations. Consequently, the downscaling model preserves the statistical properties of the coarse ESM input: the downscaled distributions are neither shifted nor skewed relative to their bias-corrected coarse counterparts, and the alignment extends to the tails of the distributions. As a result, any temporal changes or variations in the distribution shape present in the coarse input are faithfully retained in the downscaled output.

Figure 6 presents a qualitative assessment of the spatial and temporal progression of downscaled ESM simulations. The model output reproduces spatial patterns, which are, for example, consistent with geographical features, such as the Alps, and temporal patterns, like the day-night cycle, while introducing local weather dynamics consistent with the observations. Together, Figs. 5 and 6 show that the model can generate high-resolution weather trajectories that introduce complex local dynamics while preserving the value distribution of the respective ESM simulations.

## Discussion

We introduced a probabilistic approach to joint spatial and temporal downscaling of multiple variables from climate to weather scale. The presented framework revolves around a generative diffusion model, which is trained to learn an implicit representation of the dynamical patterns in reanalysis data and serves as a probabilistic emulator for the forward dynamics model. By conditioning the forward model via an observation model on climate-model output, we can sample from a posterior downscaling distribution. Each sample drawn from this posterior adheres to the established statistical relationship between climate output and fine-scale weather and avoids inconsistencies between time steps and variables, which would lead to physically implausible behavior.

Our approach aligns with a broader trend^19,41–45^ of replacing computationally expensive simulations with statistical models that emulate Earth-system dynamics. Rampal et al. ^46^ give a topical overview and discussion regarding the use of ML methods for statistical downscaling. In particular, generative-modeling techniques, such as normalizing flows^47^, GANs^18,48^, and diffusion models^19,43,49^ have been emerging as a popular model class. Our model extends existing research [for example^19,43,45,50,51^] by enforcing coherence across spatial, temporal, and variable dimensions. In particular, our predictions are sampled from a *joint* distribution, avoiding the disconnection that typically comes with sampling sequences of temporally independent states for each individual atmospheric variable. Inconsistencies between downscaled time steps and variables make predicted time series as a whole physically implausible and render the predictions unsuitable for downstream applications that require coherent estimates. We present our framework as one possible technique for such coherence-dependent applications, which could include, e.g., driving dynamical climate impact models and studies of compound events^52^, which require inter-variable coherence. A recent related method by Srivastava et al.^53^ treats the spatial and temporal domain jointly while focusing on a single variable (precipitation). For a chosen, fixed sequence length, their pipeline generates high-resolution estimates from coarse simulations by separating the task into, first, a deterministic statistical downscaling model, followed by a generative model that introduces probabilistic estimates of high-frequency patterns. A related model class is conditional weather generators^8,46^ [Ref. ^4^, Chapter 13], which require substantial expertise and resources to implement. By training an unconditional generative model, we differ from previous approaches that integrate the conditioning directly into the training process^19,45,49^. In contrast to Harder et al. ^42^, our method enables soft and uncertainty-aware constraints *post-training* through posterior inference. Hess et al. ^43^ exploit the iterative-denoising aspect of diffusion models and initialize the generative process at only partially perturbed target state. This allows conditioning the model on large-scale information, which the model enhances by replacing the remaining noise with local patterns. Notably, this notion of “conditioning” lacks a clear probabilistic interpretation and is thus fundamentally different from how we use the term throughout this work. Further, existing work focuses on mapping between different reanalysis data sets^42,54^, whereas we explicitly developed a model that allows mapping different climate scenarios to finely resolved time series that predict local weather patterns. Our trained model can be readily reused for mapping from different coarse predictors to the target resolution without retraining, simply by adapting the observation model and input data accordingly. Diffusion bridges have been proposed for unpaired downscaling of fluid dynamics, though their application to climate-model downscaling remains to be demonstrated^44^.

The intended scope of our work is to demonstrate the score-based data assimilation framework by Rozet and Louppe^23^ as an elegant and flexible technique for probabilistic downscaling and, prospectively, for other inference problems in the context of atmospheric dynamics. More than outperforming existing per-variable and per-time-step downscaling methods, or establishing our downscaling framework as a new, universally favored approach, we aim to provide a framework for downstream applications that

- require spatio-temporally coherent time series,
- consider multiple variables jointly,
- benefit from flexibly encoding additional prior knowledge about the relation between input and output through the post-training conditioning.

The comprehensive feature set offered by our model complicates direct comparisons with existing methods. Nevertheless, we demonstrate that, according to standard statistical distance metrics, our framework maintains competitive performance when evaluated per time step and per variable against benchmark approaches (see Supplementary Table 1). Further, spectral density comparisons between predictions and reanalysis data are presented in Supplementary Section 2, and a concise assessment of predictive skill for wind-power generation is provided in Supplementary Section 4. Supplementary Fig. 11 demonstrates that the model uses learned relationships between the jointly processed variables. Looking ahead, we anticipate that the expanded capabilities of the presented framework will broaden its applicability to a wider range of use cases requiring coherent, high-resolution climate information. Additionally, the modular structure of our approach (Fig. 1) enables the straightforward integration of further, potentially complex, domain-specific knowledge via the observation model in future work. For example, established functional relationships between predicted climate variables and external forcings could be incorporated without retraining the generative model. This modularity allows the trained score model to serve as a reusable foundation for subsequent studies. The diffusion model, including trained weights, and the implementations of the methods and experiments are provided at https://github.com/schmidtjonathan/Climate2Weather.

The main limitation of the presented framework is the increased computational cost that comes with coherent predictions. While we have extended the original method by Rozet and Louppe^23^ in terms of scalability, enabling it to process substantially longer trajectories, the temporal and spatial extent of the study is still limited by computational demands. Due to the simultaneous processing of the entire state space, memory requirements remain a limiting factor. This study limits itself to a small region (inspired by Langguth et al. ^50^) with a diverse range of orography to establish and validate the methodological framework. However, we believe that long-range teleconnections that affect the sub-region under study are, to some extent, reflected in the regional predictions, given that the training data has been drawn from a Europe-wide reanalysis dataset. This argument is visually supported by embedding high-resolution predictions into a larger spatial context of reanalysis data in Supplementary Section 3 (Supplementary Figs. 3 to 6). We conclude that the technique is likely most useful for smaller study regions, for which highly accurate predictions of local dynamics are desired.

In summary, our framework enables joint spatial and temporal downscaling of multiple climate variables, producing coherent, high-resolution scenarios from coarse climate simulations. By decoupling the learning of dynamical patterns from the conditioning on new inputs, the model offers a flexible and efficient tool for inference tasks in meteorology and climate science. This approach facilitates the use of long-term climate projections for local impact studies across multiple timescales.

## Methods

### Data

We analyze four interrelated atmospheric variables, namely the zonal (uas) and meridional (vas) components of near-surface (10 m) wind speeds, surface (2 m) air temperature (tas), and sea level pressure (psl), due to their crucial importance for understanding atmospheric dynamics. They evolve on different spatial and temporal scales, enabling insights into the performance of downscaling methods across different scales.

For training (2006–2013) and evaluation (2014) of the model, we use data from the Consortium for Small-Scale Modeling Reanalysis 6 (COSMO-REA6) data set, a high-resolution reanalysis product for the European domain developed by the German Weather Service (Deutscher Wetterdienst; DWD)^55^ with a spatial resolution of ~6 km and hourly temporal resolution. It serves as the ground truth observational dataset in the perfect-predictor evaluation^37^ of the presented methodological framework. The COSMO-REA6 data contains errors for some variables and lacks some observations for the years prior to 2006. In this work, only data from 2006 onwards were used.

We apply our downscaling model to historical model runs from two established general circulation models (GCMs) that are part of the sixth phase of the Coupled Model Intercomparison Project (CMIP6)^3^, namely the higher-resolution earth system model (ESM) of the Max–Planck Institute (MPI-ESM1.2-HR)^56^ and the high-resolution configuration of the third Hadley Centre Global Environment Model in the global coupled configuration 3.1 (HadGEM3-GC3.1-LM)^57^. Both models have an ~100 km spatial and 6-hourly temporal resolution.

We restrict our analysis to a spatial subregion that is oriented at the benchmark region proposed by Langguth et al. ^50^. The region includes parts of Germany, Switzerland, Austria, and the Czech Republic (6°*E*−16°*E*, 46°*N*−52°*N* (see Supplementary Fig. 1) which results in 128 × 128 grid points per time point for the reanalysis dataset. We use data for the period 2006–2014, the time range in which the reanalysis and the historical GCM runs overlap. The GCM data is spatially re-gridded to a rotated latitude-longitude grid using bilinear interpolation to match the coordinates and grid type of the reanalysis dataset. As a pre-processing step, we apply a quantile-mapping procedure to the GCM data. This mitigates biases by adjusting the value distribution of the GCM to align better with the distribution of the reanalysis product. For this, we use the quantile-mapping implementation provided by the python-cmethods^58^ Python package with default parameters.

### Model

We approach statistical downscaling as a Bayesian-inference problem^59,60^: An uncertain estimate for an unknown quantity (here: high-resolution weather) is obtained by first formulating a prior distribution that encodes known, assumed, or learned properties of the unknown quantity. The prior defines the output space of the prediction model. Through an observation model (or “likelihood”), the prior is conditioned on the available information (here: coarsely simulated climate output) to yield the posterior distribution. The posterior ideally captures the uncertainty that is both inherent in the prior and that arises from incomplete information and model mismatch.

For inference in the context of dynamical systems, it is useful to formulate a prior model that somehow represents a mechanistic system that we assume to underlie the unknown dynamics. The core of the presented methodological framework is a score-based diffusion model (DM)^20,21,61–63^, which constitutes the prior model of the framework. DMs are an instance of generative models that involve training a deep neural network on a finite set of data points, which can then be used to generate new samples from the underlying data distribution. The basic formulation of DMs involves two main components. Firstly, a forward *diffusion process* transports the data distribution to a known, tractable distribution, such as a standard normal distribution^20,21^. In this process, structured data points (like RGB images, weather states, etc.) are successively perturbed until all signals are entirely replaced by noise. The pivotal insight is that a certain class of diffusion processes is known to have a reverse counterpart, providing a generative process that successively refines samples from the noise distribution into structured data. The reversal of the diffusion process requires access to the *score function*, which can be understood as a function that, for any given degree of perturbation during the reverse diffusion process, separates noise from the signal to remove it. An exemplary such de-noising process for sequences of meridional wind speeds is visualized in Supplementary Fig. 10. In the following, we will give a brief overview of the general framework and the extensions to it necessary to obtain the results presented in this work.

The considered class of diffusion processes is Gauss–Markov processes that are the solution to linear stochastic differential equations of the form^64^

2 $${\rm{d}}X(\tau )={\rm{F}}X(\tau ){\rm{d}}\tau +{\rm{L}}{\rm{d}}W(\tau ),\quad X(0) \sim {\mathcal{N}}\left({X}_{0},\,{\Sigma }_{0}\right).$$

The state *X*(*τ*) at *τ* = 0 is a data point ${X}_{0} \sim {\mathcal{D}}$ selected from a data set ${\mathcal{D}}$. The data point is successively perturbed through the diffusion process and thus loses all structure as *τ* → T. Note that *τ* is sometimes referred to as “time”, which does not mean physical time but rather a continuous degree of perturbation of the initial state. Steps in physical time will later be denoted in the subscript, e.g., *X*_1:*L*_(*τ*) ≔ (*X*_1_(*τ*), …, *X*_*L*_(*τ*)) will denote a time series of length *L* perturbed according to *τ*. The drift F and dispersion L define functional properties of the forward process, which is driven by Brownian motion *W*(*τ*). At a final time step T (often T = 1), the process converges to a Gaussian distribution such that $X({\rm{T}}) \sim {\mathcal{N}}\left(0,\Pi \right)$, where the final-step covariance *Π* depends on the choice of F and L and is often modeled to be the identity matrix. From a result by Anderson^65^, the reverse process of Eq. (2) is known and given as

3 $${\rm{d}}X(\tau )=\left[{\rm{F}}X(\tau )-{\rm{L}}{{\rm{L}}}^{\top }\mathop{\underbrace{{\nabla }_{X(\tau )}\log {p}_{\tau }(X(\tau ))}}\limits_{{\rm{score}}\,{\rm{function}}}\right]{\rm{d}}\tau +{\rm{L}}{\rm{d}}\overleftarrow{W}(\tau ),\quad X({\rm{T}}) \sim {\mathcal{N}}\left(0,\Pi \right).$$

This process is driven by reverse Brownian motion $\overleftarrow{W}(\tau )$ and depends on the gradient of the log-marginal density, called the *score*, of the diffused state *X*(*τ*) at every perturbation-time point *τ*. We call Eq. (3) the *generative process,* as it allows sampling unseen data points from the data distribution simply by

- Sampling a vector of independent Gaussian noise $X({\rm{T}}) \sim {\mathcal{N}}\left(0,\Pi \right)$
- Numerically simulating Eq. (3) backwards through time, starting from *X*(T) and ending at a generated sample *X*(0).

There is much existing and active research regarding the sampling algorithm used in step 2, which, in general, is slower and more complex when comparing diffusion models to other methods from the generative-model class^66–68^. Hess et al. ^43^ employ a recent extension to the diffusion-model framework that allows single-step sampling. This work uses a standard technique that solves an ordinary differential equation related to the marginal distribution of Eq. (3)^21,22,63^, following the original proposal by Rozet and Louppe^23^.

In practice, the score of *p*_*τ*_(*X*(*τ*)) is not accessible and has to be estimated. It is common practice to train a noise-dependent neural network *s*_*θ*_(*X*(*τ*), *τ*) on a finite set of samples from the data distribution to approximate the true, intractable score function. Most practical approaches optimize a de-noising score-matching objective^20–22,62,63,69^. In light of this, simulating Eq. (3) is commonly perceived as iteratively de-noising the initial Gaussian random state *X*(T) and successively refining it to a noise-free data point. For spatial data, a time-conditioned U-Net^20,70^ architecture has proven effective in modeling the score function. The U-Net architecture consists of multiple levels, each containing blocks of convolutional layers and skip connections. Along these levels, the spatial dimensionality of the input data is first successively reduced and then increased again. This encoder-decoder structure results in a bottleneck layer that forces the model to extract a limited set of meaningful features from the input. At each level, skip connections between equal-resolution blocks are introduced to facilitate the learning task by maintaining information throughout the encoding and decoding process. This work uses a U-Net architecture with a total number of around 72 million parameters, including a self-attention mechanism at the 8 × 8-bottleneck layer^20,27^.

The framework used in the presented experiments leverages the work by Rozet and Louppe^23^ that extends the basic DM framework, enabling robust sampling from a posterior distribution and generating arbitrary-length sequences of spatiotemporally coherent state trajectories. The idea behind score-based data assimilation (SDA) is to separate an inference task into two parts:

I. Learning a generative model that generates spatiotemporal patterns from the target output space and
II. Conditioning the prior model using a functional or statistical constraint (the observation model) that defines the specific inference task.

This separation aligns with the idea of Bayesian inference, in which a posterior estimate is obtained from combining a prior model (I.) with an observation model (II.) through conditioning. A high-level introduction of both parts follows below. For more details regarding the score-based data assimilation method, an extensive evaluation, and the corresponding code base, we refer to the original publication by Rozet and Louppe^23^.

The generative model (I.) constitutes the prior in the present Bayesian-inference approach to statistical climate downscaling. As described above, the diffusion model requires a trained score model *s*_*θ*_(*X*(*τ*), *τ*) to iteratively refine an initial random-noise sample into a generated data point (cf. Eq. (3)). In our case, the generative model outputs uninformed sequences of high-resolution weather patterns. To generate sequential data, Rozet and Louppe^23^ propose to learn a de-noising model on short fixed-length time windows. During training, the time window is flattened into the channel dimension of the convolutional neural network that represents the score model *s*_*θ*_. This allows the model to learn correlations between different variables at different points in time. In the sampling routine, each point in time is de-noised by computing the score in the context of a surrounding temporal context window of size *w* = 2*k* + 1. Rozet and Louppe^23^ argue that many dynamical systems are (approximately) Markovian, and in order to predict the current state, it is sufficient to regard the Markov blanket of order *k* around the current time step, instead of the entire trajectory. This yields output dynamics that are temporally coherent without the need for training the network on long sequences, which would not only significantly increase the computational complexity but also fix the desired sequence length *L* as a pre-determined hyperparameter, greatly limiting flexibility. Through an intricate convolution-like routine, the windows are assembled into trajectories of arbitrary length *L*. The corresponding pseudo-code is taken from Rozet and Louppe^23^ and given in Algorithm 1.

#### Algorithm 1

Composing the sequential score function *s*_*θ*_(*X*_1:*L*_(*τ*), *τ*)
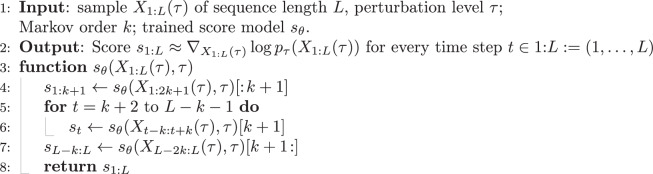


For our experiments, we require scaling the original SDA framework to allow for the prediction of very long time horizons with thousands of steps. Unfortunately, sampling a spatiotemporally coherent trajectory in the described manner comes at an increased computational cost and, in particular, significant memory demands since the entire state trajectory must be held in memory. To make the approach scale to predicting 1-hourly weather dynamics for multiple years, we implement the spatiotemporal score function Algorithm 1 as a massively parallelized convolution operation through time that efficiently manages the available memory—optionally on multiple devices. This extension of the original implementation has proven indispensable for the problem scale. The corresponding Python implementation is provided at https://github.com/schmidtjonathan/Climate2Weather.

The generative model described above can generate random sequences of weather-like patterns. One can think of it as a “physical prior” in that it represents a statistical model for the general spatial and temporal patterns observed in high-resolution weather dynamics. Note that the physical laws are only implicitly represented by this model, through learning it from existing simulations, and no explicit mechanistic laws are encoded in the model. To tackle the downscaling task specifically, we have to inform the prior model about the climate constraints. To this end, we introduce the conditioning mechanism, which allows us to impose the constraints provided by the coarse ESM simulations onto the prior model.

We proceed to describe the conditioning mechanism (II.). While the unconditioned diffusion model generates samples *p*(*X*(0)), the goal is to sample from a posterior *p*(*X*(0)∣*Y*), instead. We denote the high-resolution output with *X*(0) and the coarse input with *Y*. Here, we include the noise level *τ* of the sample *X*(*τ*) into the notation (in the parentheses), as it will become relevant in this section. Recall that *X*(0) is the generated, noise-free sample. An observation model *p*(*Y*∣*X*(0)) relates the coarse input *Y* to *X*(0). We consider a Gaussian observation model

4 $$p(Y| X(0))={\mathcal{N}}(Y;h(X(0)),{\rm{R}}),$$

with an observation operator *h*(*X*(0)) that can be a linear or non-linear function^23,71^ and observation- or sensor noise defined by a positive-definite matrix R. In our case, *h* selects from the high-resolution state *X* every 6 h and computes an area average in space (see Eq. 1). For our experiments, the observation noise R is selected by running a simple random search on a range of plausible values. To accelerate model selection, we chose R based on the model’s downscaling performance on a 2-day window. The resulting observation model is used in all presented experiments. Sampling from *p*(*X*(0)∣*Y*) amounts to replacing the score function in Eq. (3) by a posterior score function ${\nabla }_{X(\tau )}\log {p}_{\tau }(X(\tau )| Y)$. The posterior score is obtained through Bayes’ rule by noting that

5 $${\nabla }_{X(\tau )}\log p(X(\tau )| Y)={\nabla }_{X(\tau )}\log {p}_{\tau }(X(\tau ))+{\nabla }_{X(\tau )}\log p(Y| X(\tau )),$$

The normalization constant is not dependent on *X* and thus vanishes when taking the gradient. The prior score is modeled with the parametric score model *s*_*θ*_(*X*(*τ*), *τ*). Note that the likelihood term *p*(*Y*∣*X*(*τ*)) is defined on the perturbed state *X*(*τ*) and has to be approximated to be compatible with the observation model *p*(*Y*∣*X*(0)). For details, we refer the reader to [ref. ^71^, Section 3.1], who establishes the concept of “Diffusion Posterior Sampling” and to its extension by Rozet and Louppe^23^ (Section 3.2). Further, a mathematical description is given in Supplementary Section 6. To make the conditioning work efficiently at the problem scales considered in our experiments, we introduce an approximation in the conditioning mechanism that avoids the computation of gradients with respect to the score network. This is described and derived in Supplementary Section 6.

### Experimental setup

ESM projections are not directly paired to reanalysis data, so we begin by evaluating the generative downscaling model in an on-model setting. We set up an experimental setting that allows us to compare the model output to a ground truth to assess its predictive performance. Firstly, the model is trained on a subset (2006–2013) of the COSMO reanalysis data. A separate subset (2014) serves as a test set. Then, …

1. …from the test set, we select an evaluation period and generate artificial coarse observations that represent ESM projections. The spatial resolution is reduced by a factor of 16 × 16 and the temporal resolution by a factor of 6 (see Eq. 1). Specifically,
  - every 6th time step (hour) is selected from the ground truth,
  - a patch-wise spatial averaging operation is applied throughout the spatial region at every time step. We compute the arithmetic mean for each 16 × 16 spatial patch to yield a single spatial observation.
2. With the observations from 1., the model predicts the underlying 1-hourly, high-resolution weather dynamics lost by coarsening the data. Multiple samples are drawn.
3. The samples are compared to the reanalysis data from the same time period and evaluated. Variations between the samples provide structured uncertainty.

As a second step, ESM (CMIP 6) simulations replace the artificially coarsened reanalysis data for the final experiment. Instead of artificially spatiotemporally subsampled reanalysis data, we condition the score model on two different ensembles from the CMIP 6 data set: the MPI-HR and the HadGEM3 runs. The experimental setup is adopted exactly from the on-model experiments, aside from the ESM conditioning information and the extended considered time horizon of one full year. Concretely, we downscale the ESM simulations for the considered spatial patch from January 1, 06:00 A.M. until December 31, 06:00 A.M. (8736 h) for the year 2014, increasing the spatial resolution from 8 × 8 (~100 km) to 128 × 128 (~6 km) grid points. For the four high-resolution variables, this corresponds to a total of 4 × 8 736 × (128 × 128) = 572,522,496 predicted values given conditioning information that is coarser by a factor of (16 × 16) × 6 = 1536. For this specific experiment, we used four NVIDIA A-100 GPUs, each of which generated two predictions in parallel. Generating 1-year sample of hourly downscaled predictions for the 128 × 128-node region takes around 2 h on a single NVIDIA A-100 device.

## Supplementary information

Supplementary information

## Data Availability

The reanalysis data were taken from the COSMO REA-6 reanalysis product^55^. The CMIP6 data^3^ was downloaded from the Earth System Grid Federation (ESGF) at https://esgf-node.llnl.gov/projects/cmip6/.

## Code availability

The code (in Python) for model training and evaluation, data processing, and for reproducing all presented experiments and figures contained in this manuscript, is available at https://github.com/schmidtjonathan/Climate2Weather. For the qunatile-mapping bias adjustment, the Python library python-cmethods^58^ was used. Part of the plots were generated using the Cartopy library^72^. The neural-network model and training are implemented using the PyTorch library^73^.

## References

[1] Bauer, P., Thorpe, A. & Brunet, G. The quiet revolution of numerical weather prediction. *Nature* **525**, 47–55 (2015). 26333465 10.1038/nature14956

[2] Moon, J.-Y. et al. Earth’s future climate and its variability simulated at 9 km global resolution. *EGUsphere* **2024**, 1–46 (2024).

[3] Eyring, V. et al. Overview of the Coupled Model Intercomparison Project Phase 6 (CMIP6) experimental design and organization. *Geosci. Model Dev.* **9**, 1937–1958 (2016).

[4] Maraun, D. & Widmann, M. *Statistical Downscaling and Bias Correction for Climate Research* (Cambridge University Press, 2018).

[5] Laprise, R. Regional climate modelling. *J. Comput. Phys.* **227**, 3641–3666 (2008).

[6] Tapiador, F. J., Navarro, A., Moreno, R., Sánchez, J. L. & García-Ortega, E. Regional climate models: 30 years of dynamical downscaling. *Atmos. Res.* **235**, 104785 (2020).

[7] Risser, M. D. et al. Is bias correction in dynamical downscaling defensible? *Geophys. Res. Lett.* **51**, e2023GL105979 (2024).

[8] Wilks, D. S. Multisite downscaling of daily precipitation with a stochastic weather generator. *Clim. Res.* **11**, 125–136 (1999).

[9] Baño Medina, J., Manzanas, R. & Gutiérrez, J. M. Configuration and intercomparison of deep learning neural models for statistical downscaling. *Geosci. Model Dev.* **13**, 2109–2124 (2020).

[10] Baño-Medina, J., Manzanas, R. & Gutiérrez, J. M. On the suitability of deep convolutional neural networks for continental-wide downscaling of climate change projections. *Clim. Dyn.* **57**, 2941–2951 (2021).

[11] Balmaceda-Huarte, R., Baño-Medina, J., Olmo, M. E. & Bettolli, M. L. On the use of convolutional neural networks for downscaling daily temperatures over southern South America in a climate change scenario. *Clim. Dyn.* **62**, 383–397 (2024).

[12] Babaousmail, H., Hou, R., Gnitou, G. T. & Ayugi, B. Novel statistical downscaling emulator for precipitation projections using deep convolutional autoencoder over northern Africa. *J. Atmos. Sol. Terr. Phys.* **218**, 105614 (2021).

[13] Smith, D. et al. Robust skill of decadal climate predictions. *Npj Clim. Atmos. Sci.* **2**, 13 (2019).

[14] Doblas-Reyes, F. et al. Linking global to regional climate change. In *Proc. Climate Change 2021: The Physical Science Basis. Contribution of Working Group I to the Sixth Assessment Report of the Intergovernmental Panel on Climate Change*, (eds. Masson-Delmotte, V. et al.) 1363–1512 (Cambridge University Press, 2021).

[15] Morelli, S., Effenberger, N., Schmidt, L. & Ludwig, N. Climate data selection for multi-decadal wind power forecasts. *Environ. Res. Lett.* **20**, 044032 (2025).

[16] Chen, D. et al. Framing, context, and methods. In *Proc. Climate Change 2021: The Physical Science Basis. Contribution of Working Group I to the Sixth Assessment Report of the Intergovernmental Panel on Climate Change*, (eds. Masson-Delmotte, V. et al.) 147–286 (Cambridge University Press, Cambridge, 2021).

[17] Jain, S. et al. Importance of internal variability for climate model assessment. *npj Clim. Atmos. Sci.* **6**, 68 (2023).

[18] Hess, P., Drüke, M., Petri, S., Strnad, F. M. & Boers, N. Physically constrained generative adversarial networks for improving precipitation fields from earth system models. *Nat. Mach. Intell.* **4**, 828–839 (2022).

[19] Aich, M. et al. Conditional diffusion models for downscaling & bias correction of Earth system model precipitation. *arXiv* https://arxiv.org/abs/2404.14416 (2024).

[20] Ho, J., Jain, A. & Abbeel, P. Denoising diffusion probabilistic models. In *Proc. Advances in Neural Information Processing Systems*, (eds. Larochelle, H., Ranzato, M., Hadsell, R., Balcan, M. & Lin, H.) Vol. 33, 6840–6851 (Curran Associates, Inc., 2020).

[21] Song, Y. et al. Score-based generative modeling through stochastic differential equations. In *Proc. International Conference on Learning Representations* 37799–37812 (ACM Digital Library, 2021).

[22] Song, J., Meng, C. & Ermon, S. Denoising diffusion implicit models. In *Proc. International Conference on Learning Representations* (IEEE, 2021).

[23] Rozet, F. & Louppe, G. Score-based data assimilation. In *Proc.**Advances in Neural Information Processing Systems*, (eds. Oh, A. et al.) Vol. 36, 40521–40541 (Curran Associates, Inc., 2023).

[24] Kingma, D. P. & Welling, M. Auto-encoding variational bayes. In *Proc. 2nd International Conference on Learning Representations, ICLR 2014, Banff, AB, Canada, April 14-16, 2014, Conference Track Proceedings* (eds. Bengio, Y. & LeCun, Y.) (Open review net, 2014).

[25] Goodfellow, I. et al. Generative adversarial nets. In *Proc. Advances in Neural Information Processing Systems*, (eds. Ghahramani, Z., Welling, M., Cortes, C., Lawrence, N. & Weinberger, K.) Vol. 27 (Curran Associates, Inc., 2014).

[26] Dinh, L., Krueger, D. & Bengio, Y. NICE: non-linear independent components estimation. *ICLR Workshop* (2015).

[27] Dhariwal, P. & Nichol, A. Q. Diffusion models beat GANs on image synthesis. In *Proc. Advances in Neural Information Processing Systems* (eds. Beygelzimer, A., Dauphin, Y., Liang, P. & Vaughan, J. W.) (ACM Digital Library, 2021).

[28] Cao, H. et al. A survey on generative diffusion models. IEEE Transactions on Knowledge and Data Engineering, **36**, 2814–2830, 10.1109/TKDE.2024.3361474 (2024).

[29] Koo, H. & Kim, T. E. A Comprehensive survey on generative diffusion models for structured data. *arXiv* https://arxiv.org/abs/2306.04139 (2023).

[30] Rozet, F. & Louppe, G. Score-based data assimilation for a two-layer quasi-geostrophic model. *Machine Learning and the Physical Sciences Workshop, NeurIPS* (2023)*.*

[31] Klein, W. H., Lewis, B. M. & Enger, I. Objective prediction of five-day mean temperatures during winter. *J. Atmos. Sci.* **16**, 672–682 (1959).

[32] Maraun, D. et al. Precipitation downscaling under climate change: recent developments to bridge the gap between dynamical models and the end user. *Rev. Geophys.* **48**, 10.1029/2009RG000314 (2010).

[33] Lessig, C. et al. AtmoRep: a stochastic model of atmosphere dynamics using large scale representation learning. *arXiv* https://arxiv.org/abs/2308.13280 (2023).

[34] Lam, R. et al. Learning skillful medium-range global weather forecasting. *Science* **382**, 1416–1421 (2023). 37962497 10.1126/science.adi2336

[35] Price, I. et al. Probabilistic weather forecasting with machine learning. *Nature* **637**, 84–90 (2025). 39633054 10.1038/s41586-024-08252-9 PMC11666454

[36] Yang, S. et al. Generative assimilation and prediction for weather and climate. *arXiv* https://arxiv.org/abs/2503.03038 (2025).

[37] Maraun, D. et al. Value: a framework to validate downscaling approaches for climate change studies. *Earth’s. Future* **3**, 1–14 (2015).

[38] Dawid, A. P. Present position and potential developments: some personal views: statistical theory: the prequential approach. *J. R. Stat. Soc. Ser. A* **147**, 278–292 (1984).

[39] Volosciuk, C., Maraun, D., Vrac, M. & Widmann, M. A combined statistical bias correction and stochastic downscaling method for precipitation. *Hydrol. Earth Syst. Sci.* **21**, 1693–1719 (2017).

[40] Maraun, D. Bias correction, quantile mapping, and downscaling: revisiting the inflation issue. *J. Clim.* **26**, 2137–2143 (2013).

[41] Adewoyin, R. A., Dueben, P., Watson, P., He, Y. & Dutta, R. TRU-NET: a deep learning approach to high resolution prediction of rainfall. *Mach. Learn.* **110**, 2035–2062 (2021).

[42] Harder, P. et al. Hard-constrained deep learning for climate downscaling. *J. Mach. Learn. Res.* **24**, 1–40 (2023).

[43] Hess, P., Aich, M., Pan, B. & Boers, N. Fast, scale-adaptive and uncertainty-aware downscaling of Earth system model fields with generative machine learning. *Nat. Mach. Intell.* **7**, 363–373 (2025).

[44] Bischoff, T. & Deck, K. Unpaired downscaling of fluid flows with diffusion bridges. *Artif. Intell. Earth Syst.* **3**, e230039 (2024).

[45] Schmidt, L. & Ludwig, N. Wind power assessment based on super-resolution and downscaling–a comparison of deep learning methods. *arXiv preprint* https://arxiv.org/abs/2407.08259 (2024).

[46] Rampal, N. et al. Enhancing regional climate downscaling through advances in machine learning. *Artif. Intell. Earth Syst.* **3**, 230066 (2024).

[47] Groenke, B., Madaus, L. & Monteleoni, C. Climalign: unsupervised statistical downscaling of climate variables via normalizing flows. In *Proc. 10th International Conference on Climate Informatics*, 60–66 (2020).

[48] Harris, L., McRae, A. T. T., Chantry, M., Dueben, P. D. & Palmer, T. N. A generative deep learning approach to stochastic downscaling of precipitation forecasts. *J. Adv. Model. Earth Syst.* **14**, e2022MS003120 (2022). 10.1029/2022MS003120 PMC9788314 36590321

[49] Tomasi, E., Franch, G. & Cristoforetti, M. Can AI be enabled to perform dynamical downscaling? A latent diffusion model to mimic kilometer-scale COSMO5.0_CLM9 simulations. *Geosci. Model Dev.* **18**, 2051–2078 (2025).

[50] Langguth, M. et al. A benchmark dataset for meteorological downscaling. In *Proc. International Conference on Learning Representations* (ICLR, 2024).

[51] Addison, H., Kendon, E., Ravuri, S., Aitchison, L. & Watson, P. A. Machine learning emulation of precipitation from km-scale regional climate simulations using a diffusion model. *arXiv preprint* https://arxiv.org/abs/2407.14158 (2024).

[52] Zscheischler, J. et al. Future climate risk from compound events. *Nat. Clim. Change* **8**, 469–477 (2018).

[53] Srivastava, P. et al. Precipitation downscaling with spatiotemporal video diffusion. In *Proc. Thirty-eighth Annual Conference on Neural Information Processing Systems* https://openreview.net/forum?id=hhnkH8ex5d (2024).

[54] Winkler, C., Harder, P. & Rolnick, D. Climate variable downscaling with conditional normalizing flows. *arXiv* https://arxiv.org/abs/2405.20719 (2024).

[55] Bollmeyer, C. et al. Towards a high-resolution regional reanalysis for the European CORDEX domain. *Q. J. R. Meteorol. Soc.* **141**, 1–15 (2015).

[56] Müller, W. A. et al. A higher-resolution version of the Max Planck Institute Earth System Model (mpi-esm1. 2-h). *J. Adv. Model. Earth Syst.* **10**, 1383–1413 (2018).

[57] Andrews, M. B. et al. Historical simulations with HadGEM3-GC3. 1 for CMIP6. *J. Adv. Model. Earth Syst.* **12**, e2019MS001995 (2020).

[58] Schwertfeger, B. T. btschwertfeger/python-cmethods: v2.3.0 10.5281/zenodo.12168002 (2024).

[59] Gelman, A. et al. *Bayesian Data Analysis* 3rd edn (Chapman and Hall/CRC, 2013).

[60] Bishop, C. M. *Pattern Recognition and Machine Learning*. Information Science and Statistics 1st edn (Springer, 2006).

[61] Sohl-Dickstein, J., Weiss, E., Maheswaranathan, N. & Ganguli, S. Deep unsupervised learning using nonequilibrium thermodynamics. In *Proc. 32nd International Conference on Machine Learning*, vol. 37 of *Proceedings of Machine Learning Research*, (eds. Bach, F. & Blei, D.) 2256–2265 (PMLR, 2015).

[62] Kingma, D., Salimans, T., Poole, B. & Ho, J. Variational diffusion models. In *proc. Advances in Neural Information Processing Systems*, (eds Ranzato, M., Beygelzimer, A., Dauphin, Y., Liang, P. & Vaughan, J. W.) Vol. 34, 21696–21707 (Curran Associates, Inc., 2021).

[63] Karras, T., Aittala, M., Aila, T. & Laine, S. Elucidating the design space of diffusion-based generative models. In *Advances in Neural Information Processing Systems*, (eds. Koyejo, S. et al.) Vol. 35, 26565–26577 (Curran Associates, Inc., 2022).

[64] Särkkä, S. & Solin, A. *Applied Stochastic Differential Equations* (Cambridge University Press, 2019).

[65] Anderson, B. D. Reverse-time diffusion equation models. *Stoch. Process. Their Appl.* **12**, 313–326 (1982).

[66] Zhang, Q. & Chen, Y. Fast sampling of diffusion models with exponential integrator. In *Proc. The Eleventh International Conference on Learning Representations* (2023).

[67] Song, Y., Dhariwal, P., Chen, M. & Sutskever, I. Consistency models. *Proceedings of the 40th International Conference on Machine Learning*, Vol. 202, 32211–32252 (PMLR, 2023).

[68] Zheng, H., Nie, W., Vahdat, A., Azizzadenesheli, K. & Anandkumar, A. Fast sampling of diffusion models via operator learning. In *Proc. 40th International Conference on Machine Learning*, (eds. Krause, A. et al.) Vol. 202 of *Proceedings**of Machine Learning Research*, 42390–42402 (PMLR, 2023).

[69] Vincent, P. A connection between score matching and denoising autoencoders. *Neural Comput.* **23**, 1661–1674 (2011). 21492012 10.1162/NECO_a_00142

[70] Ronneberger, O., Fischer, P. & Brox, T. U-net: Convolutional networks for biomedical image segmentation. In *Proc. Medical Image Computing and Computer-Assisted Intervention (MICCAI)*, Vol. 9351 of *LNCS*, 234–241 (Springer, 2015).

[71] Chung, H., Kim, J., McCann, M. T., Klasky, M. L. & Ye, J. C. Diffusion posterior sampling for general noisy inverse problems. In *Proc. The Eleventh International Conference on Learning Representations* (2023).

[72] Met Office. *Cartopy: a cartographic Python library with a Matplotlib interface*. Exeter, Devon http://scitools.org.uk/cartopy (2010–2015).

[73] Paszke, A. et al. Pytorch: an imperative style, high-performance deep learning library https://arxiv.org/abs/1912.01703 (2019).

