# Supplementary material for: A Generative Framework for Probabilistic, Spatiotemporally Coherent Downscaling of Climate Simulation
Source: NPJ Clim Atmos Sci. 2025 Jul 18;8(1):270. doi: 10.1038/s41612-025-01157-y (PMC12270901; doi:10.1038/s41612-025-01157-y)
Supplement: Supplementary file 1 — Supplementary information [file 41612_2025_1157_MOESM1_ESM.pdf]

A Generative Framework for Probabilistic, Spatiotemporally Coherent Downscaling of Climate Simulation

Supplementary Text and Figures

Jonathan Schmidt[*,1,2], Luca Schmidt[1,2], Felix M. Strnad[1,2], Nicole Ludwig[1,2], Philipp Hennig[1,2]

[1]: University of Tübingen, Tübingen, Germany
[2]: Tübingen AI Center, Tübingen, Germany
[*]: Corresponding author. Email: jonathan.schmidt@uni-tuebingen.de

Supplementary Section 1 Spatial region: coarse and fine grid

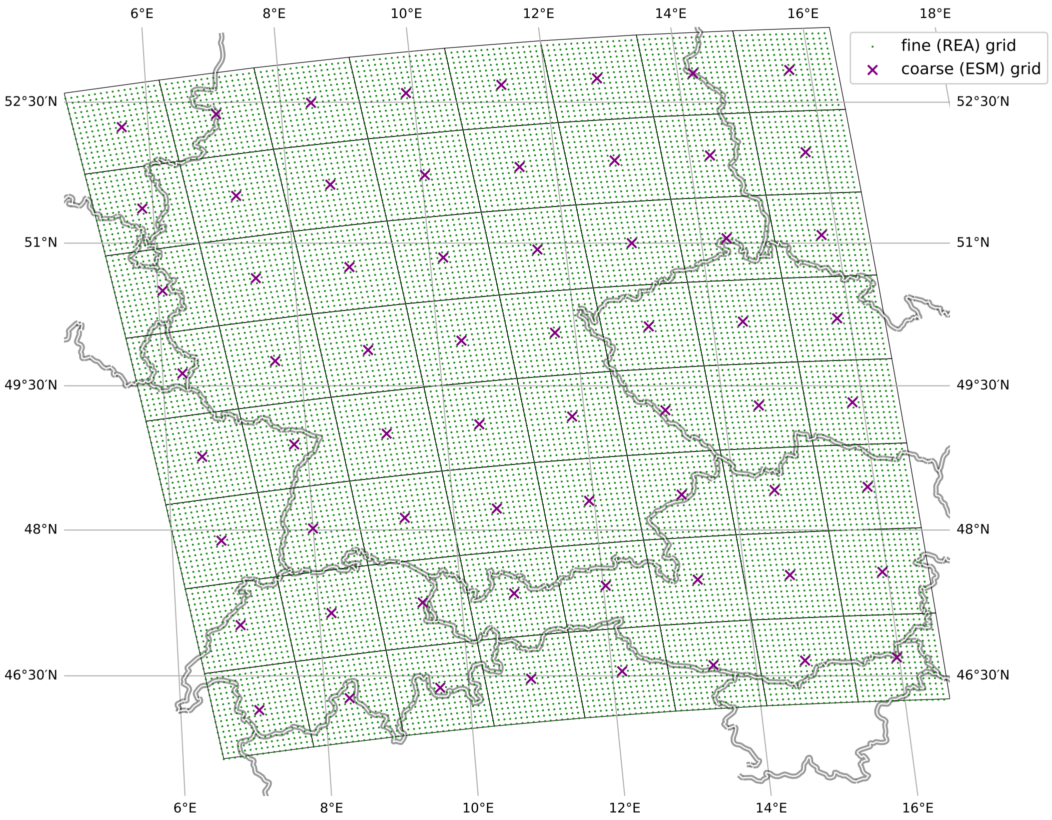

Supplementary Figure 1: This plot shows the spatial region considered in this study. The coarse 8×8-node grid is marked with purple crosses. The fine 128×128-node grid is marked with green dots. Each coarse-grid node lies in the center of a corresponding 16×16-patch of high-resolution grid nodes. Both grids span exactly the same area.

Supplementary Section 2 Spatial patterns on multiple scales

To assess the performance of the downscaling model across multiple spatial length scales, we show that the radially averaged power spectral densities (RAPSD) [1] of the predictions align with the reanalysis data in Supplementary Figure 2. The RAPSD is computed by averaging the power spectrum over all directions of the same wavenumber in Fourier space. The quantity is commonly used in the context of weather dynamics, especially when estimating precipitation [2, 3, for example]. We use the open-source `pysteps` package by Pulkkinen et al. [4] to compute the RAPSD. The reported RAPSD values are averages over the considered time period.

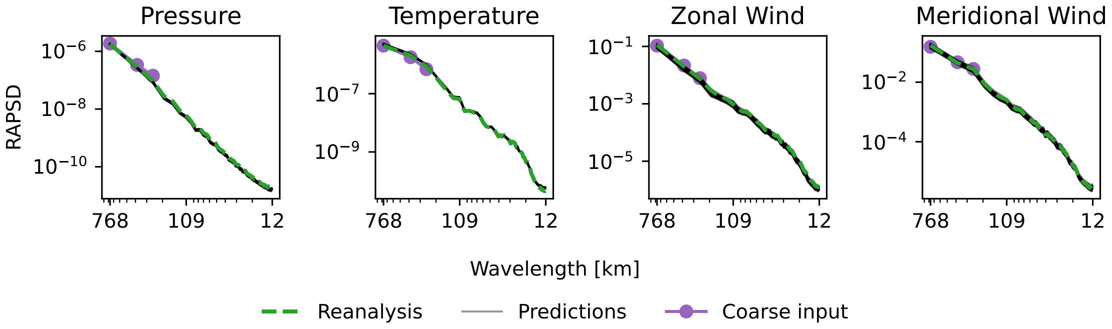

(a) RAPSD for reanalysis data, coarse input, and predicted fine-grained reanalysis data.

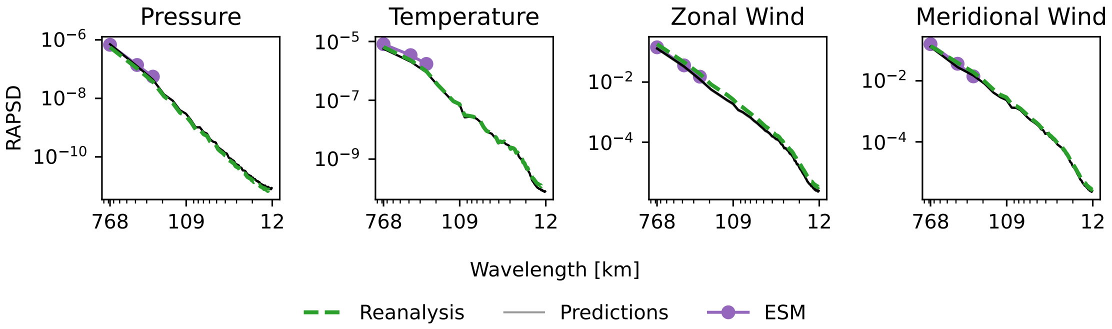

(b) RAPSD for reanalysis data, CMIP6 simulations, and downscaled CMIP6 simulations.

Supplementary Figure 2: This plot shows the RAPSD for reanalysis data, coarse input, and the corresponding downscaled predictions. Subplot (a) mirrors the experimental setup of the on-model experiment that predicts high-resolution reanalysis data during the cyclone "Friederike" in January 2018. Subplot (b) covers the CMIP6 downscaling setup as described in the Methods section.

Supplementary Section 3 Embedding the predicted region in an extended spatial context

We embed the high-resolution predictions during the cyclone "Friederike" (c.f. Figure 4) into a larger spatial context in order to learn about how long-distance interconnections between the studied spatial region and its surroundings are captured by the model. We argue that it is likely that our statistical downscaling model is able to capture the global connectedness of weather dynamics that are contained in the reanalysis data it is trained on. Concretely, Supplementary Figures 3 to 6 visualize the spatiotemporal dynamics of downscaled samples, reanalysis data, and conditioning input. Thereby, the spatial region is extended beyond the one considered in this study. Reanalysis data is used to fill in the regions outside of the predicted patch. Clearly visible or implausible transitions at the edges of the patch would indicate that the model predictions do not align with the surrounding spatial context. We find, however, that there is a smooth transition from outside the predicted area to its interior for the generated high-resolution predictions. The generated local dynamics seamlessly fit into the more surrounding context of reanalysis data.

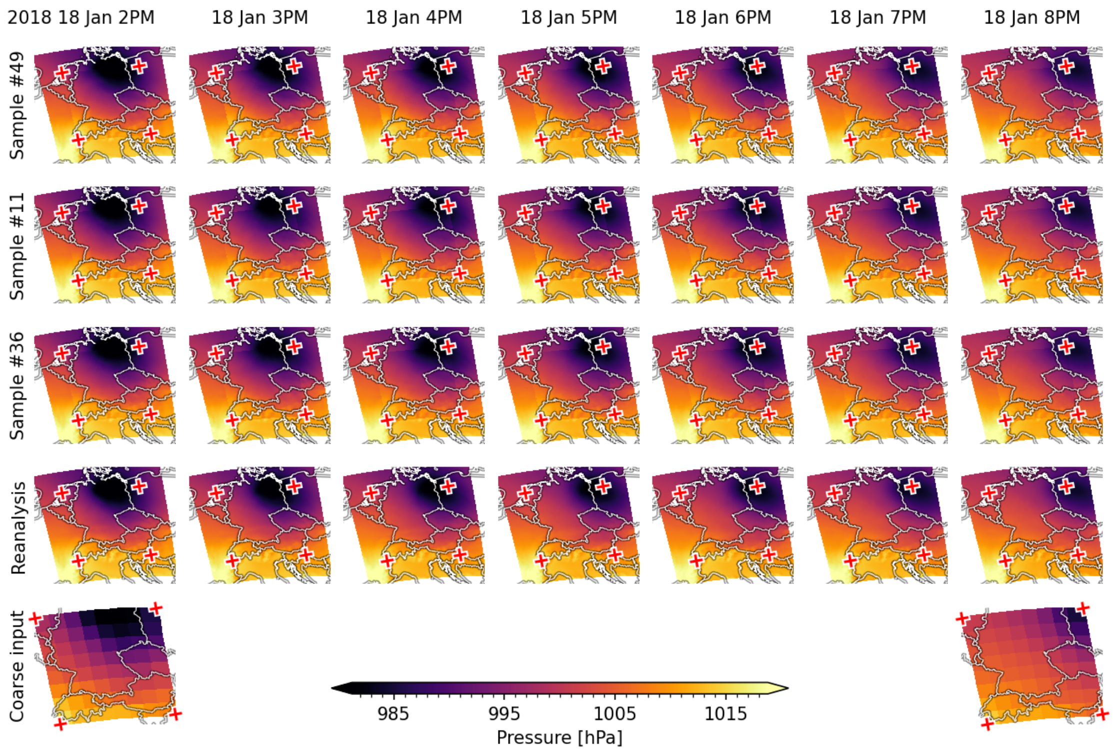

Supplementary Figure 3: Predicting mean sea-level pressure during the cyclone "Friederike" (c.f. experiment above). Outside of the prediction range, which lies in the region indicated by red crosses, reanalysis data is filled in to give an impression as to how the predictions fit into more global dynamics.

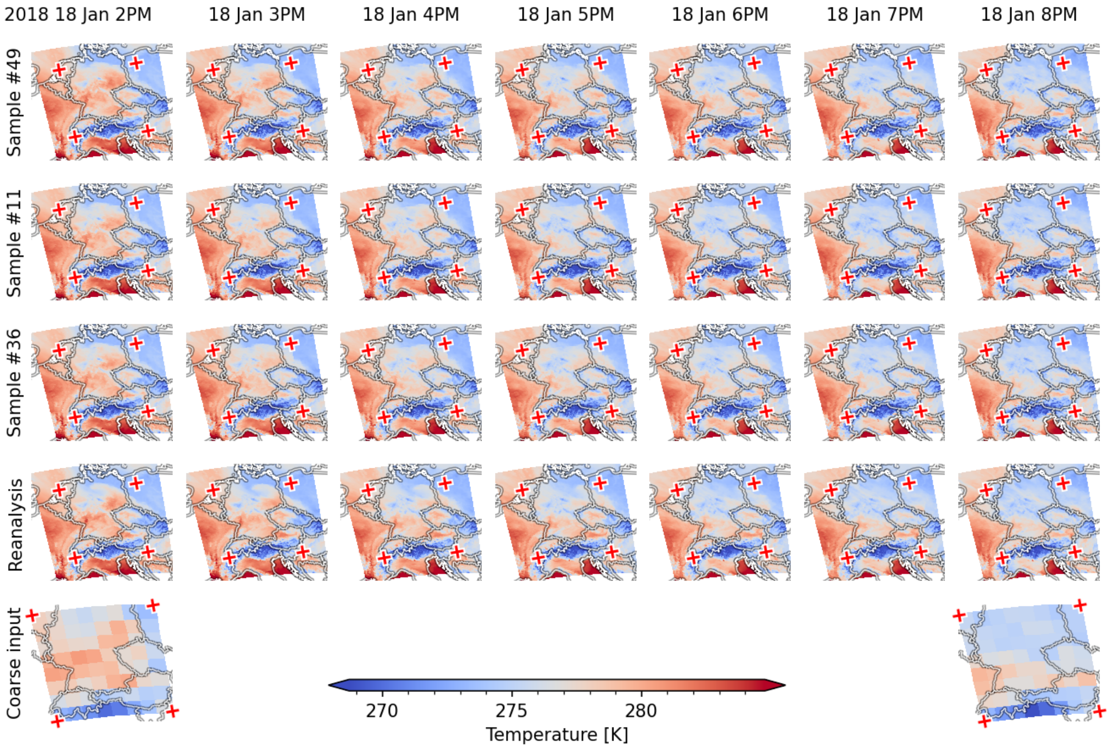

Supplementary Figure 4: Predicting surface air temperature during the cyclone "Friederike" (c.f. experiment above). Outside of the prediction range, which lies in the region indicated by red crosses, reanalysis data is filled in to give an impression as to how the predictions fit into more global dynamics.

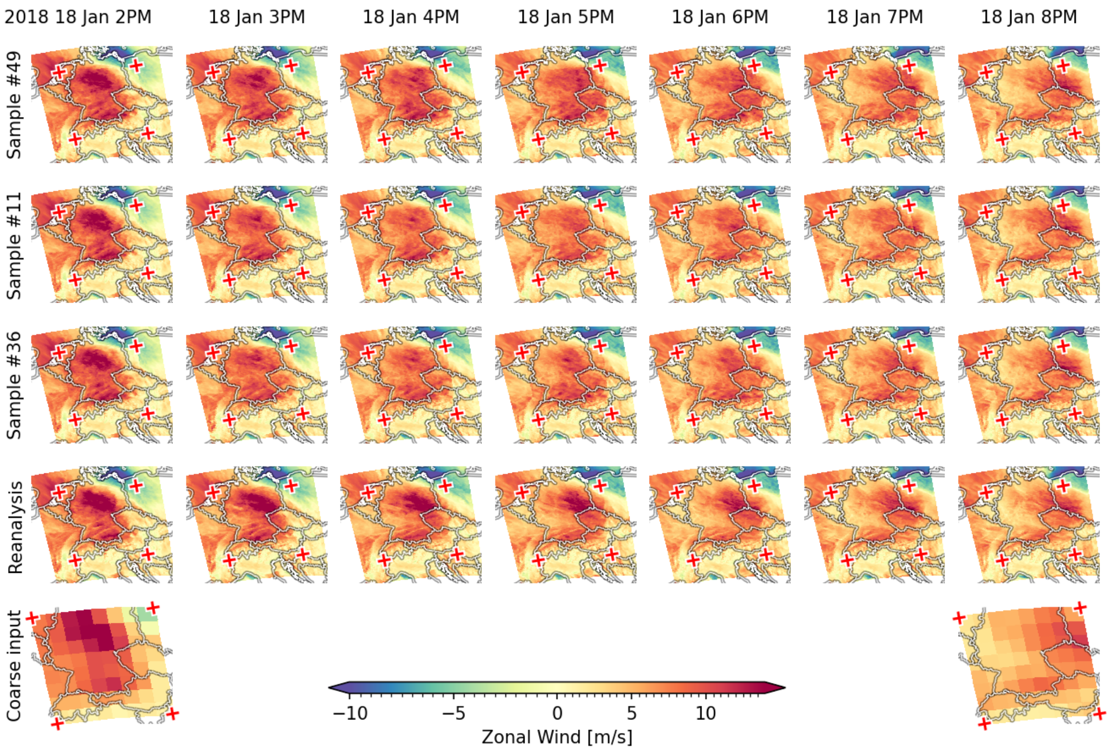

Supplementary Figure 5: Predicting zonal wind speed during the cyclone "Friederike" (c.f. experiment above). Outside of the prediction range, which lies in the region indicated by red crosses, reanalysis data is filled in to give an impression as to how the predictions fit into more global dynamics.

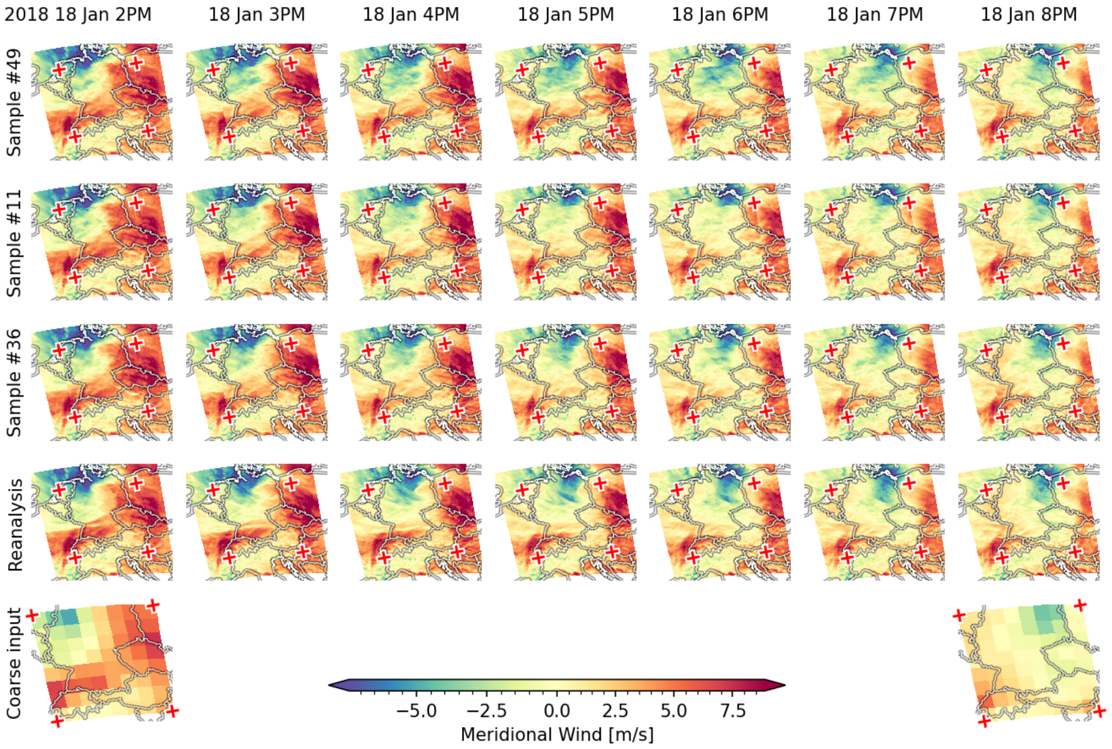

Supplementary Figure 6: Predicting meridional wind speed during the cyclone "Friederike" (c.f. experiment above). Outside of the prediction range, which lies in the region indicated by red crosses, reanalysis data is filled in to give an impression as to how the predictions fit into more global dynamics.

Supplementary Section 4 Wind power prediction

A crucial motivation for spatiotemporal downscaling climate simulations to the weather scale is downstream tasks that require future local weather patterns. We provide an exemplary evaluation of the estimated generated wind powers as computed from the CMIP6 simulation, the downscaled predictions, and the reanalysis data. We find that, when computing the spatial average of generated wind powers, the wind-speed and wind-power predictions of the ESM are matched by the downscaled samples in distribution (Supplementary Figure 7). Comparing single locations reveals that the ESM locally sometimes over-predicts or under-predicts the wind power generated following the reanalysis data. For both cases, we consistently find that our model corrects the respective over- and under-estimation for multiple randomly selected locations. We compare estimated densities of the wind-speed values (Supplementary Figure 7 **a**) and use this distribution to derive the amount of wind power generated from the respectively predicted wind speeds (Supplementary Figure 7 **b**). For that, we first compute the wind-power curve for a range of wind-speed values (0m/s to 30m/s), using the open-source package `windpowerlib` [5] and an arbitrary wind-turbine model (turbine type: "E-115/3000", hub-height: 100m). Then, we weigh this power curve with the density of predicted wind speeds to obtain the predicted wind power for each wind speed, taking into account how likely this wind speed value is to occur in the predictions. Finally, we compare the accumulated generated wind power over time. We show the comparison between two pairs of locations in Supplementary Figure 8.

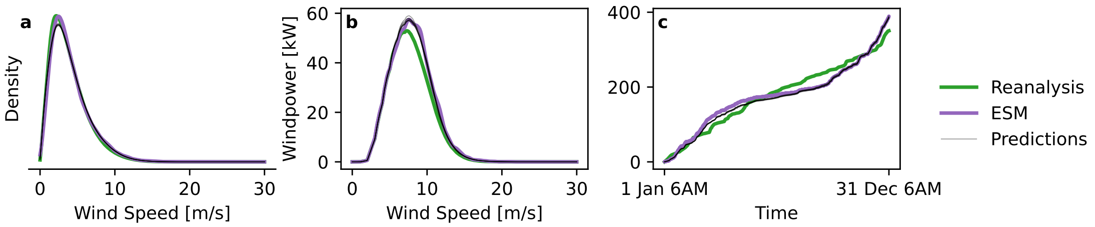

Supplementary Figure 7: **Estimated generated wind power over time, averaged over the spatial domain.** This plot analyzes the local wind-speed predictions via the generated wind-power derived from the wind speeds. The full spatial region is considered over the year 2014. Subplot **a** visualizes a kernel-density estimate of the aggregated wind speeds for reanalysis data (green), ESM simulations (purple), and downscaled predictions (black). In **b**, we plot the generated wind power for the entire range of wind speeds from 0m/s to 30m/s, weighted by the density of the estimated wind speeds from **a**. We use a wind-power curve of the form Carrillo et al. [6, Fig. 1]. This estimates the wind power that is generated for the respective wind speeds and accounts for the frequency at which these wind speeds occur. Finally, subplot **c** shows the cumulative sum of the generated wind powers over time. All wind-power values are normalized by the number of time steps on the respective temporal grids to align the wind-power scales. From the black lines aligning closely with the purple line, we conclude that the downscaled predictions preserve the aggregated wind-power generation as simulated by the ESM.

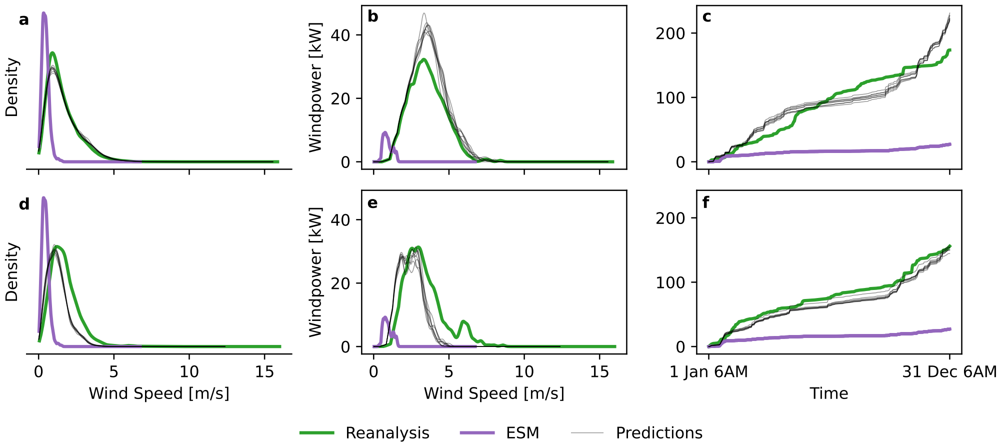

(a) Compare generated windpower at two distinct locations that share a single node on the coarse ESM grid. In this instance, the ESM underpredicts the generated windpower.

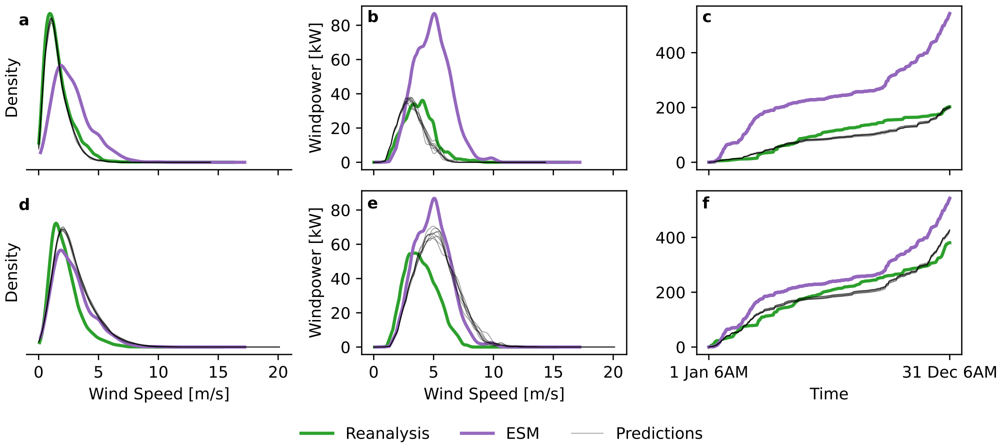

(b) Compare generated windpower at two distinct locations that share a single node on the coarse ESM grid. In this instance, the ESM overpredicts the generated windpower.

Supplementary Figure 8: **Local estimates of wind power over time.** This plot investigates two pairs of locations that are each constrained by a single spatial node on the coarse climate grid. The layout of each row of the subplots (a) and (b) mirrors Supplementary Figure 7; each row corresponds to a single location on the fine-resolution grid, respectively.

Supplementary Section 5 Quantitative evaluation

We first apply quantile-mapping to bias-correct the climate patches. These frames are used as input for the different downscaling algorithms. Our model is compared to the following two benchmark approaches.

Benchmark 1: Interpolation The first benchmark consists of a bias-correction step, followed by bi-linearly interpolating the coarse data to a finer spatial grid. This two-step procedure is called Bias Correction Spatial Disaggregation (BCSD) [7] and is both simple and prominently used in the downscaling literature. The reanalysis data is first remapped to match the coarse resolution of the climate model grid. The bias correction step then uses quantile mapping applied to the entire spatial domain. To account for seasonal variations, our implementation uses a moving average (with a window size of 25 days) centered around each calendar day when computing the quantiles. The temporal pooling ensures robust statistics by considering similar days from the seasonal cycle together.

Benchmark 2: Conditioned frame-to-frame diffusion model While our approach leverages a score-based approach, i.e., all states/trajectories are generated simultaneously in a non-autoregressive manner, we compare it to a conventional frame-by-frame modeling approach using a denoising diffusion implicit model (DDIM) [8]. We use a time-conditional U-Net backbone, incorporating residual blocks and self-attention layers. The conditioning is then performed by bi-linearly upsampling the bias-corrected climate data to match the high-resolution data and concatenating them along the channel dimension. To predict the middle frame, we condition on a sequence of three frames [9].

Metrics We use an approximate (sliced) two-dimensional Wasserstein distance [10] as a metric to compare two high-dimensional probability distributions, defined as

$$\mathrm{W}(P_{\mathrm{pred}}, P_{\mathrm{ref}}) = \inf_{\gamma \in \Pi(P_{\mathrm{pred}}, P_{\mathrm{ref}})} \mathbb{E}_{(x,y)\sim\gamma}[\|x-y\|_1], \tag{1}$$

where $\Pi(P_{\mathrm{pred}}, P_{\mathrm{ref}})$ is the set of couplings, that is, probability distributions whose marginals are P_{pred} and P_{ref}. This variant makes the traditional Wasserstein distance computationally feasible for high-dimensional probability distributions [10]. In the sliced variant, the high-dimensional data is projected onto a one-dimensional line for which a one-dimensional Wasserstein distance can be efficiently computed. This slicing process is performed repeatedly for multiple different slices, and the result is averaged to obtain a reliable metric.

The Mean Energy Log Ratio (MELR) is used to evaluate the preservation of potentially highly varying physical patterns in the downscaled spatial patches. The MELR is a metric that is derived from the radially averaged power spectral density (Section Supplementary Section 2) as

$$\mathrm{MELR} = \sum_k \left| \log \left(\frac{E_{\mathrm{pred}}(k)}{E_{\mathrm{ref}}(k)} \right) \right|, \tag{2}$$

where the energy of the predicted field $E_{\mathrm{pred}}(k)$ is compared to that of the reference field $E_{\mathrm{ref}}(k)$.

We use the structural similarity index measure (SSIM) [11], which takes into account perceptual properties of local structures when quantifying the similarity between two spatial data points. The

SSIM is defined by sliding a window $W_k(x,y)$ of size $k \times k$ along the spatial data point that computes for two $k \times k$-patches x and y

$$W_k(x,y) = \frac{(2\mu_x\mu_y + c_1)(2\sigma_{x,y} + c_2)}{(\mu_x^2 + \mu_y^2 + c_1)(\sigma_x^2 + \sigma_y^2 + c_2)}, \tag{3}$$

where μ_x, σ_x and μ_y, σ_y are the respective average and standard-deviations of the values in the respective patches x and y. Analogously, σ_{xy} is the covariance between the patches. Finally, c_1 and c_2 are constants to make the computation more robust. The SSIM takes on values between 0 and 1, whereby higher values indicate more structural similarities between the compared data points. An SSIM of 1 can only be attained when both data points are identical. In Supplementary Table 1 we report the SSIM using a window size of $k = 15$.

Supplementary Table 1: **Quantitative evaluation of downscaling methods.** We compare the performance of our model, based on score-based data assimilation (SDA), to a conditional denoising diffusion implicit model (DDIM) and to bias-correction spatial disaggregation (BCSD). We report the sliced Wasserstein-1 distance (Sliced W1) over time, temporally averaged energy log ratio (MELR), and structural similarity index (SSIM) for the four variables: mean sea-level pressure (`psl`), surface (2m) air temperature (`tas`), surface (10m) zonal `uas`, and meridional (`vas`) wind speeds. The values are reported as mean ± standard deviation over the generated predictions. Note that, since the benchmark methods are not capable of temporal downscaling, the quantitative evaluation is performed on the 6-hourly observation grid. Even though the method proposed in this work is under the additional constraint of generating temporally consistent 1-hourly trajectories, it beats both benchmarks in most cases. Between all approaches, the best performance per metric and variable is highlighted in **bold**.

Metric \ Method	var	**SDA (ours)**	**BCSD**	**DDIM**
Sliced W1 ↓	`psl`	0.3028 ± 0.0012	$\mathbf{0.2900} \pm 0$	0.3526 ± 0.0536
	`tas`	0.3526 ± 0.0005	0.3999 ± 0	$\mathbf{0.2894} \pm 0.0999$
	`uas`	$\mathbf{0.1998} \pm 0.0015$	0.2523 ± 0	0.4055 ± 0.0586
	`vas`	$\mathbf{0.2348} \pm 0.0033$	0.2702 ± 0	0.3553 ± 0.0371
MELR ↓	`psl`	$\mathbf{1.0256} \pm 0.0031$	1.1625 ± 0	1.0874 ± 0.1022
	`tas`	$\mathbf{0.3470} \pm 0.0018$	1.1312 ± 0	0.3932 ± 0.0187
	`uas`	$\mathbf{1.4622} \pm 0.0025$	2.4141 ± 0	1.5598 ± 0.0770
	`vas`	1.4555 ± 0.0096	2.4042 ± 0	$\mathbf{1.3317} \pm 0.0664$
SSIM ↑	`psl`	0.8925 ± 0.0002	$\mathbf{0.9027} \pm 0$	0.8691 ± 0.0074
	`tas`	$\mathbf{0.8524} \pm 0.0002$	0.7066 ± 0	0.8382 ± 0.0094
	`uas`	$\mathbf{0.1351} \pm 0.0008$	0.0844 ± 0	0.1182 ± 0.0061
	`vas`	$\mathbf{0.1322} \pm 0.0009$	0.0973 ± 0	0.1185 ± 0.0068

Supplementary Section 6 Approximating the gradient of the observation model

Using Bayes' rule, we obtain the posterior score function

$$\nabla_{X(\tau)}\left[\log p_\tau(X(\tau) \mid y)\right] = \nabla_{X(\tau)}\left[\log p_\tau(X(\tau)) + \log p(Y \mid X(\tau))\right] \tag{4}$$

$$= \underbrace{\nabla_{X(\tau)}\left[\log p_\tau(X(\tau))\right]}_{\approx s_\theta(X(\tau),\tau)} + \nabla_{X(\tau)}\left[\log p(Y \mid X(\tau))\right]. \tag{5}$$

This illustrates that the conditioning mechanism, which involves only a simple addition of the gradient of the log-observation model, is independent of the trained score model. However, as detailed by Chung et al. [12], the posterior score requires relating the measurement Y to the diffused state $X(\tau)$. Chung et al. [12] propose to approximate

$$p(Y \mid X(\tau)) = \int p(Y \mid X(0), X(\tau))\; p(X(0) \mid X(\tau))\; \mathrm{d}X(0) \tag{6}$$

$$= \int p(Y \mid X(0))\; p(X(0) \mid X(\tau))\; \mathrm{d}X(0) \tag{7}$$

$$= \mathbb{E}_{X(0)\sim p(X(0)\mid X(\tau)}\left[p(Y \mid X(0))\right] \tag{8}$$

$$\approx p\left(Y \mid \hat{X}(0) := \mathbb{E}_{X(0)\sim p(X(0)\mid X(\tau)}\left[X(0)\right]\right). \tag{9}$$

Intuitively, the last step pulls the expectation into the conditioning, which is not equivalent in general, and thereby approximates $p(Y \mid X(\tau)) \approx p(Y \mid \hat{X}(0))$. The quantity $\hat{X}(0)$ is a posterior-mean estimate for the noise-free data point underlying the diffused state $X(\tau)$ at the current diffusion-time τ in the generative process. In the following, we detail how $\hat{X}(0)$ is computed.

Linear stochastic differential equations, like the diffusion process defined in Equation (2), can be equivalently formulated in terms of a discrete, linear Gaussian transition model

$$p(X(\tau + \Delta\tau) \mid X(\tau)) = \mathcal{N}\left(X(\tau + \Delta\tau); \mathrm{A}(\Delta\tau)X(\tau), \Sigma(\Delta\tau)\right), \tag{10}$$

for some time increment $\Delta\tau$. We refer to, e.g., Särkkä and Solin [13, Section 6.1] for more details on how to derive the *transition* and *process-noise covariance* functions A and Σ from the drift F and dispersion L of the diffusion process (Equation (2)). The forward diffusion process can be simulated over extended time ranges in a single step via sampling from the Gaussian transition density

$$X(\Delta\tau) \mid X(0) = \mathrm{A}(\Delta\tau)X(0) + \Sigma^{\frac{1}{2}}(\Delta\tau)\epsilon, \quad \epsilon \sim \mathcal{N}(0, 1), \tag{11}$$

which is an equivalent formulation of Equation (10) for the case $\tau = 0$, i.e., starting from the noise-free data point [14, 15, 8]. The same is not possible in the reverse direction, which would make the generation process trivial. Unfortunately, the backwards transition model associated with Equation (3) is Gaussian only for infinitesimally small time decrements $\Delta\tau \to 0$ [16, 17], which leads to sampling quality increasing with the number of steps used for simulating the generative process. However, it is possible to estimate the posterior mean $\hat{X}(0)$ based on the current score estimate

$s_\theta(X(\tau), \tau)$. By re-arranging Equation (11) we note that

$$X(0) = \mathrm{A}^{-1}(\tau)\left(X(\tau) - \Sigma^{\frac{1}{2}}(\tau)\epsilon\right) \tag{12}$$
$$= \mathrm{A}^{-1}(\tau)\left(X(\tau) + \nabla_{X(\tau)}\left[p_\tau(X(\tau))\right]\Sigma(\tau)\right) \tag{13}$$
$$\approx \mathrm{A}^{-1}(\tau)\left(X(\tau) + s_\theta(X(\tau), \tau)\Sigma(\tau)\right) \tag{14}$$
$$= \hat{X}(0). \tag{15}$$

The first equality uses the direct correspondence between ϵ and the de-noising score function of Equation (10) [14, 18]. Since the true score, like ϵ, is not known while generating a new data point, it is in the next step approximated with our parametric score model s_θ. A more detailed derivation can be found for the scalar case ($A, \Sigma \in \mathbb{R}$) in Chung et al. [12].

With that, we established the necessary background regarding the conditional model, in order to describe the approximation to the conditioning mechanism that is used in this work. Plugging into Equations (4) and (5) the approximation from Equations (6) to (9) reveals that we have to compute the gradient

$$\nabla_{X(\tau)}\left[\log p(Y \mid \hat{X}(0))\right]. \tag{16}$$

For our purposes, we assume a Gaussian observation model (Equation (4)), which simplifies this gradient to

$$\nabla_{X(\tau)}\left[\log p(Y \mid \hat{X}(0))\right] = \nabla_{X(\tau)}\left[\log \mathcal{N}\left(Y; h(\hat{X}(0)), \mathrm{R}\right)\right] \tag{17}$$
$$= \nabla_{X(\tau)}\left[\|Y - h(\hat{X}(0))\|_{\mathrm{R}}\right], \tag{18}$$

where $\|\cdot\|_{\mathrm{R}}$ is a Mahalanobis norm in the multivariate case and a Euclidean norm in the scalar case. Let us denote the residual between conditioning information and the observed predicted signal as $r(Y, \hat{X}(0)) := Y - h(\hat{X}(0))$. Then, using the chain rule of differentiation, we obtain

$$\nabla_{X(\tau)}\left[\|r(Y, \hat{X}(0))\|_{\mathrm{R}}\right] = \nabla_{r(Y,\hat{X}(0))}\left[\|r(Y, \hat{X}(0))\|_{\mathrm{R}}\right] \cdot \nabla_{\hat{X}(0)}\left[h\left(\hat{X}(0)\right)\right] \cdot \nabla_{X(\tau)}\left[\hat{X}(0)\right]. \tag{19}$$

We plug the equality from Equations (14) and (15) into the third component of the gradient to obtain

$$\nabla_{X(\tau)}\left[\hat{X}(0)\right] = \nabla_{X(\tau)}\left[\mathrm{A}^{-1}(\tau)\left(X(\tau) + s_\theta(X(\tau), \tau)\Sigma(\tau)\right)\right] \tag{20}$$
$$= \mathrm{A}^{-1}(1 + \nabla_{X(\tau)}\left[s_\theta(X(\tau), \tau)\Sigma(\tau)\right] \tag{21}$$
$$\approx \mathrm{A}^{-1}. \tag{22}$$

The final step approximation avoids the computational demanding differentiation of the score function with respect to the perturbed state, which cuts away the majority of the computational cost in the conditioning. Altogether, the approximate conditioning term (c.f. Equation (5)), used throughout this work, is

$$\begin{aligned}\nabla_{X(\tau)}\left[\log p(Y \mid X(\tau)\right] &\approx \nabla_{X(\tau)}\left[\log p(Y \mid \hat{X}(0))\right] \\ &\approx \nabla_{r(Y,\hat{X}(0))}\left[\|r(Y, \hat{X}(0))\|_{\mathrm{R}}\right] \cdot \nabla_{\hat{X}(0)}\left[h\left(\hat{X}(0)\right)\right] \cdot \mathrm{A}^{-1},\end{aligned} \tag{23}$$

which is very cheap to compute in each de-noising step.

Supplementary Section 7 Anomalies

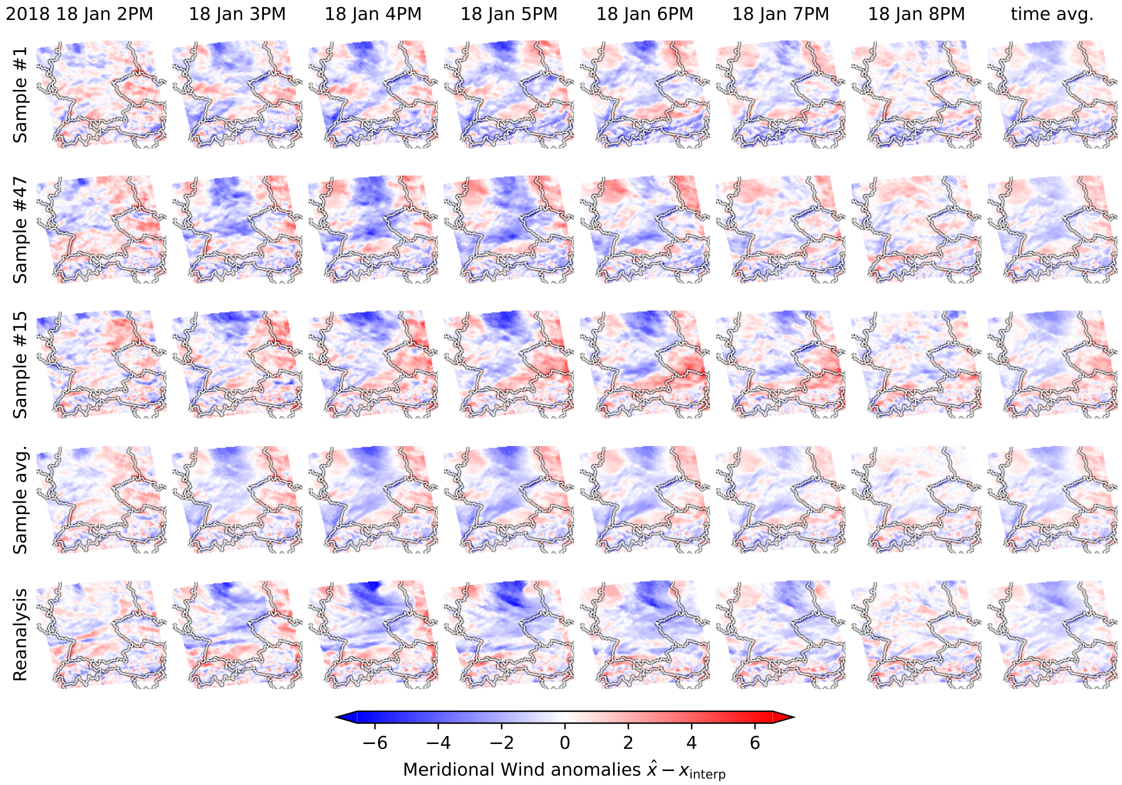

Supplementary Figure 9: **Anomalies between downscaled and interpolated versions of coarse input.** This plot supplements Figure 4. It visualizes the differences between a spatiotemporal interpolation of the coarse input x_{interp} (Figure 4, fourth row) and different fine-scale time series $\hat{x}$: 1) three samples from the proposed downscaling model (*top three rows*), 2) the average of the three samples (*fourth row*), and 3) the ground-truth reanalysis data (*bottom row*). This visualization exposes the local spatial and temporal patterns on the fine grid that are not contained in the coarse data. The rightmost column plots the corresponding temporal averages of the spatial anomalies. As in Figure 4, the downscaling model is only conditioned at 2PM (first column) and 8PM (penultimate column). Especially at those conditioning points, the local spatial patterns predicted in each sample (rows 1 through 3) are structurally similar to those in the reanalysis data (bottom row). Between the conditioning points (3PM through 7PM), the ground-truth anomalies (bottom row) expose that the temporal evolution of the cyclone is not predicted by the smooth temporal interpolation. Our model predicts spatiotemporal structure on the fine grid, adding information that cannot be trivially inferred from the coarse input.

Supplementary Section 8 Generative de-noising process

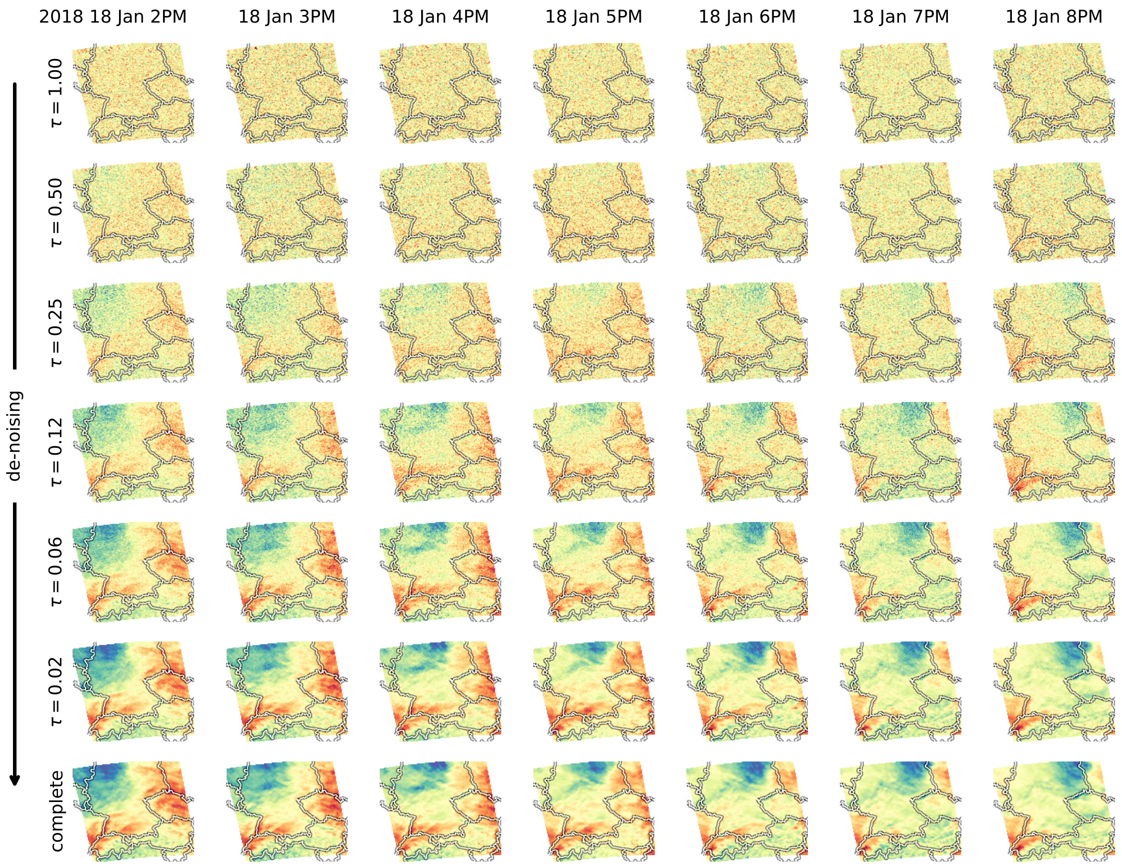

Supplementary Figure 10: **The generative process of the diffusion model.** Diffusion models learn a mapping from a tractable noise distribution ($\tau = 1$; often Gaussian noise) to the training-data distribution. Using a statistical model for the score function, which separates noise from signal, an initial random-noise sample is iteratively de-noised into a data point that lies in a region of high data density. This figure shows seven (of 256) steps of this generative process, beginning at $\tau = 1$ (*top row*; Gaussian noise) and ending at $\tau = 0$ (*bottom row*). Fine-scale features are generated by the model towards the end of the generative process. Notably, the spatial and temporal structure emerge jointly, since the score function is estimated for the entire time series.

Supplementary Section 9 Relationship between downscaled variables

This experiment demonstrates that the prediction for one variable is affected by conditioning information about the other remaining variables through inter-variable relationships that the generative model learned through training. To show this, we isolate one variable "v" of interest (here: meridional wind speeds) and denote the remaining variables (here: mean sea-level pressure, surface temperature, zonal wind speeds) as "$\neg$v". We predict four different downscaled sequences for v:

1. First, we draw from the prior. Sampling from the unconditioned generative downscaling model, yields an uninformed sequence of weather patterns.
2. Second, we draw from the generative model that is conditioned only on $\neg$v.
3. Third, we draw from the generative model that is conditioned only on v.
4. Finally, we draw from the fully conditioned generative downscaling model, providing the model with the information about all considered variables, v and $\neg$v, as is the default case in the other experiments (e.g., Figure 4).

Supplementary Figure 11 visualizes these differently-informed samples of v as spatiotemporal sequences (cf. Figure 4). A comparison between the unconditioned sample (1.; first row in Supplementary Figure 11), the different partly-conditioned predictions (2. and 3.; second and third rows in Supplementary Figure 11), and the fully-conditioned predictions (4.; fourth row in Supplementary Figure 11), demonstrates that the multivariate downscaling model has learned relationships between the variables, which it uses for generating downscaled predictions. Note that this experiment is purely diagnostic and serves to validate that the model learns and does not neglect relationships between the considered variables.

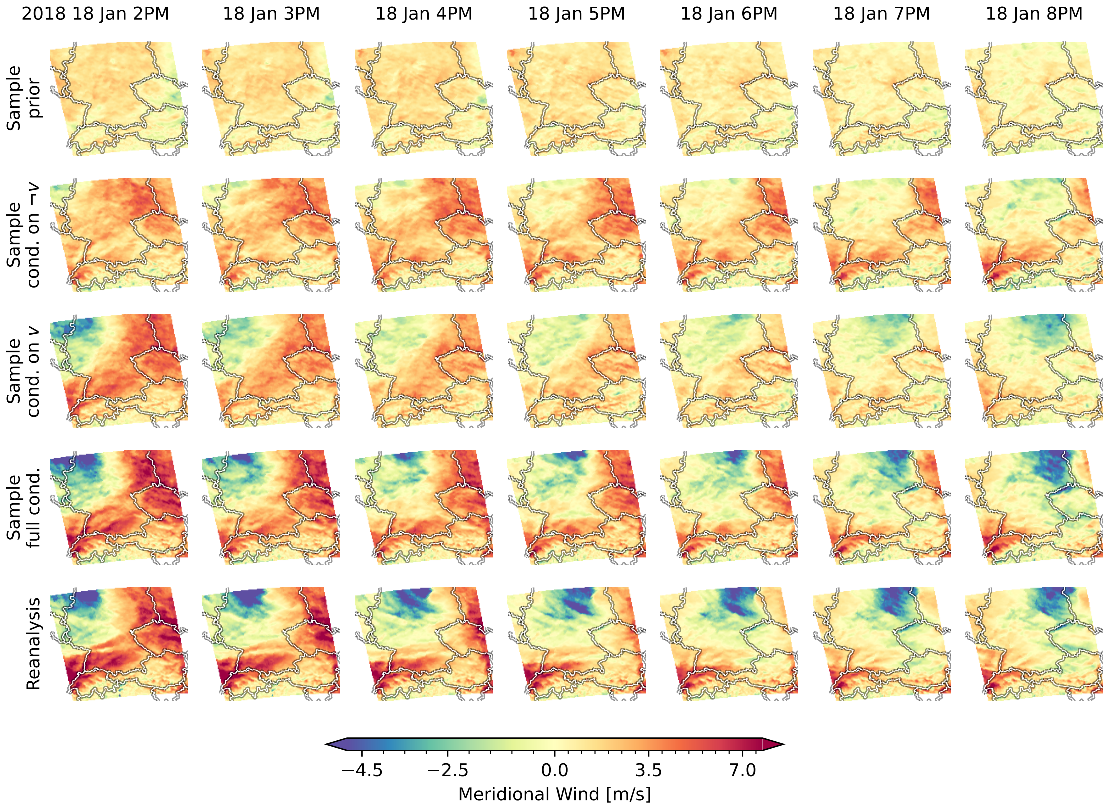

Supplementary Figure 11: **The model learns and uses relationships between variables.** The first four rows of this plot visualize four different predictions for downscaled meridional wind during a cyclone ("Friederike, January 2018). The bottom row shows the reanalysis data for comparison. For the conditioned predictions (rows 2 through 4), no information is provided to the model between the first (2:00 PM) and the last (8:00 PM) visualized time point, exactly as in Figure 4. We denote the visualized variable of interest (here: meridional wind speeds) as "v", and the other variables (here: mean sea-level pressure, surface temperature, and zonal wind speeds) as "¬v". As in Figure 4, the sign of the wind speed value defines its direction and time progresses from left to right hourly, starting 2018 January 18 at 02:00 PM and ending the same day at 08:00 PM. The *first row* shows a sample from the unconditioned generative model, which is entirely uninformed by any coarse input. The *second row* shows the downscaled v, predicted by the generative model that is only conditioned on ¬v. The *third row* shows the downscaled v, predicted by the generative model that is only conditioned on v. The *fourth row* shows the downscaled v, predicted by the generative model conditioned on all variables, v and ¬v. The plot serves to demonstrate the effect of the multivariate nature of the downscaling model. Comparing the model outputs when conditioning on different sets of variables demonstrates that the prediction of a variable v is affected by incorporating information about the other variables ¬v. This allows the conclusion that the downscaling model has learned inter-variable relationships, which it uses for prediction.

Reference

[1] Evan Ruzanski and V Chandrasekar. Scale filtering for improved nowcasting performance in a high-resolution x-band radar network. *IEEE transactions on geoscience and remote sensing*, 49 (6):2296–2307, 2011.

[2] Lucy Harris, Andrew T. T. McRae, Matthew Chantry, Peter D. Dueben, and Tim N. Palmer. A generative deep learning approach to stochastic downscaling of precipitation forecasts. *Journal of Advances in Modeling Earth Systems*, 14(10), 2022.

[3] Philipp Hess, Stefan Lange, Christof Schötz, and Niklas Boers. Deep learning for bias-correcting cmip6-class earth system models. *Earth's Future*, 11(10):e2023EF004002, 2023.

[4] S. Pulkkinen, D. Nerini, A. A. Pérez Hortal, C. Velasco-Forero, A. Seed, U. Germann, and L. Foresti. Pysteps: an open-source python library for probabilistic precipitation nowcasting (v1.0). *Geoscientific Model Development*, 12(10):4185–4219, 2019.

[5] Sabine Haas, Uwe Krien, Birgit Schachler, Stickler Bot, Velibor Zeli, Florian Maurer, Kumar Shivam, Francesco Witte, Sasan Jacob Rasti, Seth, and Stephen Bosch. wind-python/windpowerlib: Update release, February 2024. URL `https://doi.org/10.5281/zenodo.10685057`.

[6] C. Carrillo, A.F. Obando Montaño, J. Cidrás, and E. Díaz-Dorado. Review of power curve modelling for wind turbines. *Renewable and Sustainable Energy Reviews*, 21:572–581, 2013. ISSN 1364-0321.

[7] Thomas Vandal, Evan Kodra, and Auroop R Ganguly. Intercomparison of machine learning methods for statistical downscaling: the case of daily and extreme precipitation. *Theoretical and Applied Climatology*, 137:557–570, 2019.

[8] Jiaming Song, Chenlin Meng, and Stefano Ermon. Denoising diffusion implicit models. In *International Conference on Learning Representations*, 2021.

[9] Luca Schmidt and Nicole Ludwig. Wind power assessment based on super-resolution and downscaling–a comparison of deep learning methods. *arXiv preprint arXiv:2407.08259*, 2024.

[10] Sebastian Bischoff, Alana Darcher, Michael Deistler, Richard Gao, Franziska Gerken, Manuel Gloeckler, Lisa Haxel, Jaivardhan Kapoor, Janne K Lappalainen, Jakob H Macke, et al. A practical guide to sample-based statistical distances for evaluating generative models in science. *Transactions on Machine Learning Research*, 2024.

[11] Z. Wang, E.P. Simoncelli, and A.C. Bovik. Multiscale structural similarity for image quality assessment. In *The Thrity-Seventh Asilomar Conference on Signals, Systems & Computers, 2003*, volume 2, pages 1398–1402 Vol.2, 2003.

[12] Hyungjin Chung, Jeongsol Kim, Michael Thompson Mccann, Marc Louis Klasky, and Jong Chul Ye. Diffusion posterior sampling for general noisy inverse problems. In *The Eleventh International Conference on Learning Representations*, 2023.

[13] S. Särkkä and A. Solin. *Applied Stochastic Differential Equations.* Cambridge University Press, 2019.

[14] Jonathan Ho, Ajay Jain, and Pieter Abbeel. Denoising diffusion probabilistic models. In H. Larochelle, M. Ranzato, R. Hadsell, M.F. Balcan, and H. Lin, editors, *Advances in Neural Information Processing Systems*, volume 33, pages 6840–6851. Curran Associates, Inc., 2020.

[15] Yang Song, Jascha Sohl-Dickstein, Diederik P Kingma, Abhishek Kumar, Stefano Ermon, and Ben Poole. Score-based generative modeling through stochastic differential equations. In *International Conference on Learning Representations*, 2021.

[16] W Feller. On the theory of stochastic processes, with particular reference to applications. In *First Berkeley Symposium on Mathematical Statistics and Probability*, pages 403–432, 1949.

[17] Jascha Sohl-Dickstein, Eric Weiss, Niru Maheswaranathan, and Surya Ganguli. Deep unsupervised learning using nonequilibrium thermodynamics. In Francis Bach and David Blei, editors, *Proceedings of the 32nd International Conference on Machine Learning*, volume 37 of *Proceedings of Machine Learning Research*, pages 2256–2265, Lille, France, 07–09 Jul 2015. PMLR.

[18] Diederik Kingma, Tim Salimans, Ben Poole, and Jonathan Ho. Variational diffusion models. In M. Ranzato, A. Beygelzimer, Y. Dauphin, P.S. Liang, and J. Wortman Vaughan, editors, *Advances in Neural Information Processing Systems*, volume 34, pages 21696–21707. Curran Associates, Inc., 2021.
